# Hierarchical access to encoded data on DNA nanostructures using administrator and user keys

**DOI:** 10.1093/nar/gkaf835

**Published:** 2025-08-27

**Authors:** Kuiting Chen, Sisi Fan, Na Liu, Jie Song, Linqiang Pan

**Affiliations:** Key Laboratory of Image Information Processing and Intelligent Control of Education Ministry of China, School of Artificial Intelligence and Automation, Huazhong University of Science and Technology, Wuhan, Hubei 430074, China; 2nd Physics Institute, University of Stuttgart, Stuttgart 70569, Germany; Max Planck Institute for Solid State Research, Stuttgart 70569, Germany; 2nd Physics Institute, University of Stuttgart, Stuttgart 70569, Germany; Max Planck Institute for Solid State Research, Stuttgart 70569, Germany; Hangzhou Institute of Medicine, Chinese Academy of Sciences, Hangzhou, Zhejiang 310022, China; Key Laboratory of Image Information Processing and Intelligent Control of Education Ministry of China, School of Artificial Intelligence and Automation, Huazhong University of Science and Technology, Wuhan, Hubei 430074, China

## Abstract

DNA nanotechnology has shown great potential for molecular information encoding and controlled access. With the remarkable progress of DNA data storage systems, it is imperative to develop DNA-based hierarchical access techniques to manage user data. However, to our knowledge, this aspect has yet to be fully explored. Herein, we propose a data management system on the carrier of reconfigurable DNA nanostructure, which enables hierarchical access to encoded data. The system employs two types of molecular triggers—polymerase and DNA strand sets—to induce conformational changes of the nanostructure. These structural transitions allow the transformation of pre-patterned binding sites into readable data arrays. The polymerase universally triggers transformations in different carriers, functioning as a high-level administrator key, while the DNA strand set specifically transforms a particular structure, serving as a low-level user key. In a multi-user scenario, we encoded multi-format messages to illustrate data compatibility. Atomic force microscopy results demonstrated that the admin key granted access to all users’ data, while user keys provided access only to their designated data. This strategy provides a framework for hierarchical data access on DNA carriers and may inspire the development of more sophisticated DNA-based information security solutions by restricting unauthorized access and isolating user privacy.

## Graphical Abstract

**Graphical Abstract:**
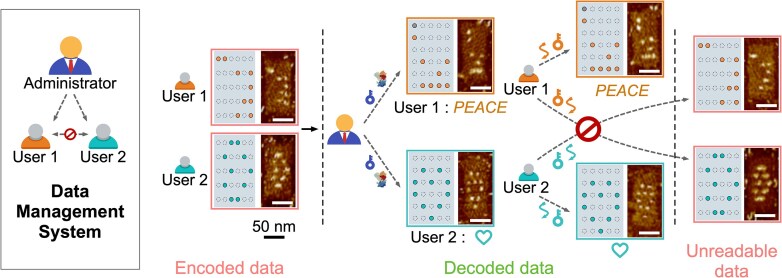

## Introduction

The secure storage and controlled access of data through encoding systems play a pivotal role in safeguarding sensitive information and personal privacy. However, traditional protocols are encountering significant challenges due to rapid advancements in high-performance computing, which may soon enable the cracking of current encryption methods within a feasible time frame using brute-force attacks. Biomolecular information encoding, which leverages complex biochemical reactions and stringent experimental protocols to manage data [1–5], has emerged as a promising alternative. By circumventing vulnerabilities inherent to electronic systems, biomolecule-based approaches offer an innovative framework for privacy-aware data handling, garnering considerable attention in recent years.

Among biomolecular strategies, DNA-based information encoding is considered one of the most promising molecular data encryption methods [6]. In 1999, Clelland *et al.* proposed a DNA-based data encoding concept [7], initiating the paradigm of converting plaintext into DNA sequences [8–10]. More recently, DNA nanostructures have been utilized to create patterned data carriers [11–15], due to their high structural versatility and precisely addressable pixels. For example, Zhang *et al.* developed a DNA origami-based method to generate data carriers with protein-binding steganographic features and theoretical key spaces up to 700 bits [11]. Their work illustrated how nanostructured DNA platforms can support confidentiality, integrity, and accessibility in molecular information systems.

The rise of DNA-based data storage systems has led to a demand for more advanced strategies to manage access permissions—similar to access control in traditional databases [16–21]. An interesting approach involves implementing a two-factor authentication strategy, incorporating both possession and knowledge factors, to control access [21]. However, existing research on access authority management primarily focuses on communication protocols and uses methods such as fluorescence reporting to demonstrate feasibility. To our knowledge, comprehensive studies that integrate access management with the full process of encoded data recovery have not yet been fully explored.

**Figure 1.**
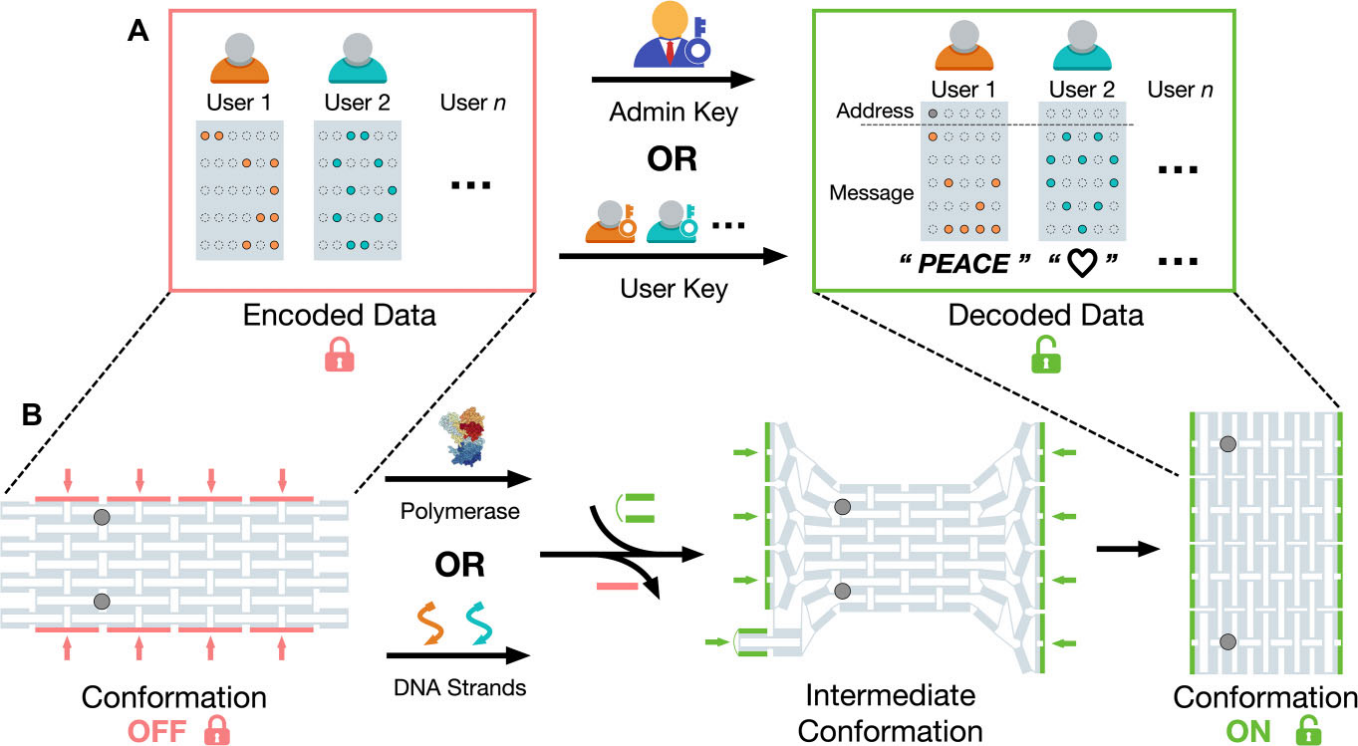
Scheme of the data management system. (**A**) Hierarchical access to encoded data using keys with different permission levels: user key and admin key. (**B**) The dynamic behavior of the DDC structure enables data decoding. Conformational transformation of the DDC enables the conversion of pre-patterned binding sites into readable data arrays, with the transformation triggers serving as the keys to the encoded data.

Herein, we proposed a DNA-based data management system to enable hierarchical access to encoded data with differential permission levels (Fig. 1A). The system utilized a reconfigurable DNA origami domino array structure as the DNA-based data carrier (DDC). Pre-arranged biomolecular labels were employed to encode information on the DDC [22–25]. Structural transformation of the DDC enables readout of the encoded information, and triggering agents acted as keys to access data. To enhance functionality, we introduced polymerase as a trigger for the reconfigurable DDC structure.

Previous studies have demonstrated the use of polymerases to modulate the conformation of DNA nanostructures. Agarwal *et al.* converted bent nanotubes into rigid conformations by generating multiple double-stranded fragments along the nanotube edges using a polymerase gap-filling reaction [26]. He *et al.* employed polymerization to reconstruct a collapsed DNA origami structure—initially assembled with only 15% of its staples—into a complete representation of a Chinese knot motif [27]. Yan *et al.* utilized Q5 polymerase to generate rigid double-stranded DNA segments at the collapsed edges of DNA domino arrays, thereby controlling their conformation [28]. In our own prior work, we achieved regionalized structural disassembly by using a few primer strands to displace dozens of staple strands [29, 30]. In these cases, the polymerase recognized the primer regions nonspecifically, bypassing the requirement for strict sequence complementarity between synthetic DNA and the binding region.

Polymerase-triggered strand displacement (PTSD) [29–31] and gap-filling reactions established a one-to-many relationship between the triggering agents and the reconfigurable DDC. This feature enabled the creation of an administrator key capable of accessing data across multiple users. In contrast, DNA strand sets, the conventional triggers of DDCs [22–25], exhibited a strict one-to-one relationship with specific nanostructures, serving as user keys to limit access to individual data and prevent unauthorized cross-access. Atomic force microscopy (AFM) images showed that the transformation efficiency of the DDC and the accuracy of data retrieval were significantly higher with the admin and matched user keys compared to mismatched user keys. The introduction of polymerase has enriched the conformational methods and application scenarios of DNA domino array structure. The proposed system supports hierarchical data access management and privacy-aware information retrieval in multi-user environments.

## Materials and methods

### Preparation of DNA origami structures

The design of the DDC structure was referenced from a previous study by Fan *et al.* [23]. Note that we used a shorter scaffold strand (M13mp18), which resulted in a smaller origami structure and data array. All DNA strands were synthesized by Sangon Biotech, Shanghai, China (see Supplementary Tables). Unmodified strands were ordered with High Affinity Purification (HAP), and biotin-modified strands were ordered with High Performance Liquid Chromatography (HPLC) purification. To generate the nanostructure, 10 nM of M13mp18 strands (Tilibit nanosystems, Garching, Germany) were mixed with staple strands (including primer strands for the PTSD reaction) at a ratio of 1:10 in 1× TE buffer (Tris, 10 mM; EDTA, 2 mM; and magnesium chloride, 12 mM; pH 8.0). The samples were then annealed for 10 h using the following thermal annealing protocol: 95°C for 5 min, from 85°C to 24°C at a rate of 10 min/°C. After annealing, the samples were purified through a 100-KDa ultracentrifugal filter (MWCO, Amicon, Millipore, Molsheim, France) to remove excess staple strands. The concentration of the purified origami was determined by the estimated extinction coefficient at 260 nm (∼1.091 × 10^8^ M^−1^ cm^−1^) [32]. In a typical sample, the concentration of purified DNA origami was ∼30 nM.

The dynamic behaviors of the DDC enabled effective data decoding. With the DDC transformed, the conformation of the data staples also shifted, resetting the data array pattern. However, the overall external shape of the DDC was largely the same before and after the transformation (a two-dimensional rectangle). Therefore, once shape-based reading rules were established, the conformational changes in the DDC could reconfigure the patterned data (Supplementary Fig. S2). For example, when the DDC transformed from a locked conformation (named OFF) to an unlocked conformation (named ON), the two-label array shifted from a short-distance to a long-distance pattern (Fig. 1B). This ability allowed us to convert the exquisitely designed patterned data from ciphertext to plaintext by transforming the DDC conformation. The transformation triggers—either polymerase or specific DNA strands—functioned as the secret keys to access the encoded data (Fig. 1B).

### Conformational transformation of nanostructures using DNA strands

To achieve conformational transformation using DNA strands, a four-fold excess of key strands (including trigger strands and invading strands) was added to the nanostructure samples. The mixtures were then incubated at 40°C for 3 h.

### Conformational transformation of nanostructures using polymerase

The samples for admin were mixed with 8 kU/l of Klenow polymerase (Thermo Fisher Scientific, Shanghai, China) in 1× Klenow buffer. The dNTP concentration was (including all four kinds of deoxyribonucleotides) ∼1–1.5 mM. Samples were incubated at 37°C for 4 h. In a typical 25 μl of transformed DDC sample, the final concentration of DNA origami structure was ∼12.5 nM.

### Information recovery with STV

To recover the steganographic information, an excess of streptavidin (STV) (Sangon Biotech, Shanghai, China) was introduced to the DDC samples (after transformation) at a concentration 10 times higher than that of the biotin sites. The samples were incubated at room temperature for 2 h to ensure efficient interaction between STV and biotin.

### AFM imaging

The mixtures were diluted to ∼1 nM (concentration of DDC) in 1× TE buffer (with 15 mM MgCl_2_) for AFM characterization. A 5 μl sample was placed onto freshly cleaved mica and allowed to sit for 5 min. Then, 15 μl of 1× TE (with 15 mM MgCl_2_) was added to the scanning area. All images were obtained by a Bruker Multimode 8 AFM (Bruker Corporation, Germany) and scanned in the “ScanAsyst in fluid” mode.

### Statistical analysis by AFM imaging

The yield of the DDC structure with expected conformation or pattern, ${{y}_{{\rm exp}}}$, was calculated using Eq. (1):


(1) \begin{eqnarray*}
{{y}_{{\rm exp}}} = \frac{M}{N},
\end{eqnarray*}

where $N$ is the total number of intact DDC structures observed in the AFM images and $M$ is the number of DDC structures with the expected conformation or pattern.

## Results

### Design of data management system

We used the previously reported DNA origami domino array structure (Supplementary Fig. S1) [23], which has been established as a DDC, to implement the data management system. The DDC was assembled from interconnected DNA anti-junctions that could propagate structural changes to neighboring junctions [22–25]. A trigger strand initiated the process by binding to one edge of an anti-junction. This change created a high-energy interface with neighboring units, prompting them to adopt the same conformation. During the transformation of the array structure, the DNA anti-junction unit underwent a conformational shift and propagated dynamic triggering to neighboring units via base stacking, ultimately reconfiguring the entire array. To support data storage, 30 staple strands from the DDC were selected to form an array (Supplementary Fig. S2). Specifically, a 5 × 5 array was designated for storing user messages, while a 1 × 5 array located above the message array was used to encode address bits (Fig. 1A). Staples in the data array were selectively replaced with biotin-modified strands. The encoded data were concealed within a customized pattern composed of these biotin-modified staples. STV was then allowed to bind to the biotinylated DNA strands, thereby revealing the encoded data pattern [11, 13, 23, 25].

The dynamic behaviors of the DDC enabled effective data decoding. With the DDC transformed, the conformation of the data staples also shifted, resetting the data array pattern. However, the overall external shape of the DDC was largely the same before and after the transformation (a two-dimensional rectangle). Therefore, once shape-based reading rules were established, the conformational changes in the DDC could reconfigure the patterned data (Supplementary Fig. S2). For example, when the DDC transformed from a locked conformation (named OFF) to an unlocked conformation (named ON), the two-label array shifted from a short-distance to a long-distance pattern (Fig. 1B). This ability allowed us to convert the exquisitely designed patterned data from ciphertext to plaintext by transforming the DDC conformation. The transformation triggers—either polymerase or specific DNA strands—functioned as the secret keys to access the encoded data (Fig. 1B).

### Conformational transformation of the DNA-based data carrier

As discussed in the pioneering study, transformation efficiency is highly related to the DNA sequences within the structure. In our system, the use of a new scaffold strand (M13mp18) resulted in anti-junction sequences distinct from those in the previously reported structure (using the p7560 scaffold). This difference likely altered the free energy landscape, favoring the maintenance of the OFF conformation (Supplementary Fig. S3). To expand the methods and enhance the efficiency of structural transformations, we first investigated the impact of edge staples on the DDC’s conformation before implementing the transformation and data access.

We designed six DDC variants, with varying number of edge strands (Supplementary Fig. S4). By analyzing the percentage of different conformations in these variants (Supplementary Figs S4–S10), we observed that a reduction in the number of edge strands increased the tendency for DDC to adopt the ON conformation. We speculated that the lack of edge strands introduced flexible single-stranded regions, resulting in collapsed structures due to entropic spring effects [26, 27], thereby favoring the ON conformation. Based on these results, we hypothesized that displacing the edge strands would likely facilitate the structural transformation of the DDC.

**Figure 2.**
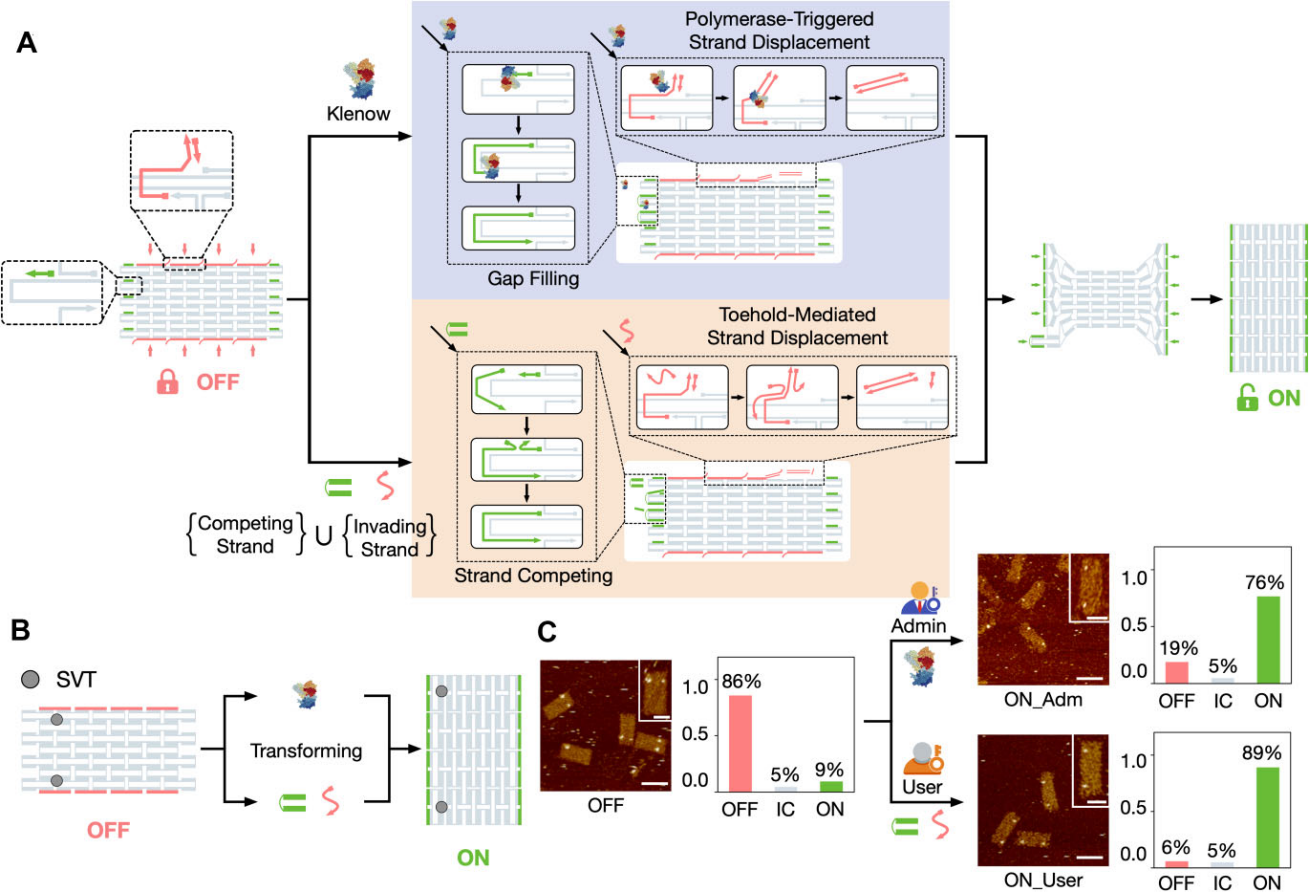
Conformational transformation of the DDC using polymerase and DNA strand set. (**A**) Schematic illustration of DDC reconfiguration. The transformation is initiated by two types of triggers: polymerase and DNA strand set. (**B**) Diagram depicting the reconfiguration of STV-marked (gray dots) DDC structures using polymerase and DNA strand set. (**C**) AFM images showing the transformation of the DDC structure. OFF, locked DDC with OFF conformation; ON, unlocked DDC with ON conformation; IC, intermediate conformation. A total of 85, 101, and 85 DDC structures are quantified for the OFF, ON_Adm, and ON_User samples, respectively. Vertical axis, fraction of the target conformation or pattern. Scale bars, 100 nm. Insets: zoomed-in AFM images. Scale bars, 50 nm.

To create the reconfigurable DDC structure (Fig. 2A), we substituted the 13 edge strands with displaceable staples featuring a 3′ overhang (red lines). Seven orthogonal sequences were designed for the overhang domains of 13 edge staples. The overhang contained a primer-binding domain and a toehold domain. The former initiated the PTSD reaction, and the latter facilitated the toehold-mediated strand displacement (TMSD) reaction. A set of single-stranded gap regions was introduced into the scaffold strand on the two short sides of the OFF conformation. Primers (green) were bound to these single-stranded scaffold regions to start the polymerase gap-filling reaction. The gap regions were perfectly complementary to the competing strand, allowing easy replacement of the green primer. This state of the DDC was regarded as the locked OFF conformation and was used for storing the encoded data (Fig. 2A, left column).

Upon treatment with Klenow polymerase, gap-filling and PTSD reactions occurred at the single-stranded scaffold regions and the displaceable edge areas, respectively (Fig. 2A, top of middle column). On the short sides, polymerization was initiated by the green-primer-bound domain, filling the single-stranded gap regions. On the long sides, the red primer initiated the PTSD reaction, leading to the removal of the edge strands from the DDC structure. To minimize undesired removal of staple strands from the inner parts of the nanostructure [27, 28], a low dose of Klenow polymerase (8 kU/l, 4 h) was used to control the reactions. When DNA strands were used to trigger the transformation of the DDC structure, a series of TMSD reactions occurred on the rectangular carrier (Fig. 2A, bottom of middle column). On the short side, competing strands (green) displaced the green primers and formed duplexes through strand competition. On the long side, invading strands (red) recognized the toeholds on the overhangs of the edge strands, gradually displacing the edge strands from the DDC and leaving single-stranded gap regions. The reaction temperature was maintained at 40°C to facilitate strand displacement. These reactions enabled the transformation of the DDC structure (Fig. 2A, right column).

Two factors were considered to drive this transformation: base stacking forces, as reported previously [22], and the inherent mechanical forces of DNA molecules. Polymerase or strand treatments generated newly formed double-stranded sides, which provided mechanical forces to straighten and extend the original short sides, while the gap regions induced a collapsing tendency along the original edges. Together, these mechanisms facilitated a predictable transformation of the DDC from the OFF to the ON conformation.

As a proof of principle, we studied the effectiveness of reconfiguring the DDC using polymerase and DNA strands (Fig. 2B). We used two STV molecules to indicate the conformation of the DDC. In the AFM image (Fig. 2C and Supplementary Figs S11–S13), two STV highlights with shorter distance indicated the OFF conformation, while two highlights with a longer distance indicated the ON conformation. By analyzing the AFM results, we found that 86% of the DDC structures remained in the OFF conformation without the addition of polymerase or DNA strands (Supplementry Table S1). After transformation, the ON conformation yields, obtained using polymerase and DNA strands, were estimated to be 76% and 89% (Supplementary Tables S2 and S3), respectively.

Since data decoding relies on the transformation of the DDC structure, we assigned administrator and user roles to the two types of transformations to establish hierarchical access. The polymerase nonspecifically recognizes the primer domain and initiates gap-filling and PTSD reactions in DNA substrates with arbitrary sequences. Thus, it could universally trigger transformations in various DDC structures, functioning as the high-level administrator key for multi-user data access. In contrast, the DNA strand set can specifically trigger the transformation of a particular DDC structure, with specificity dictated by strand sequences. This property allowed the DNA strand set to access data for a single user, thereby serving as a low-level user key.

In polymerase-triggered DDC transformation experiments, the polymerase concentration and reaction duration must be carefully optimized according to the amount of substrate structure (Supplementary Fig. S14). Similarly, the concentrations of dNTPs and STV must be tailored to the specific reaction system. While the universality of the admin key may pose potential risks to data security, the stringent experimental protocols and the need for precisely tuned reaction parameters ensure the security of the admin key during its use for accessing encoded data.

### Access encoded data using user and admin keys

**Figure 3.**
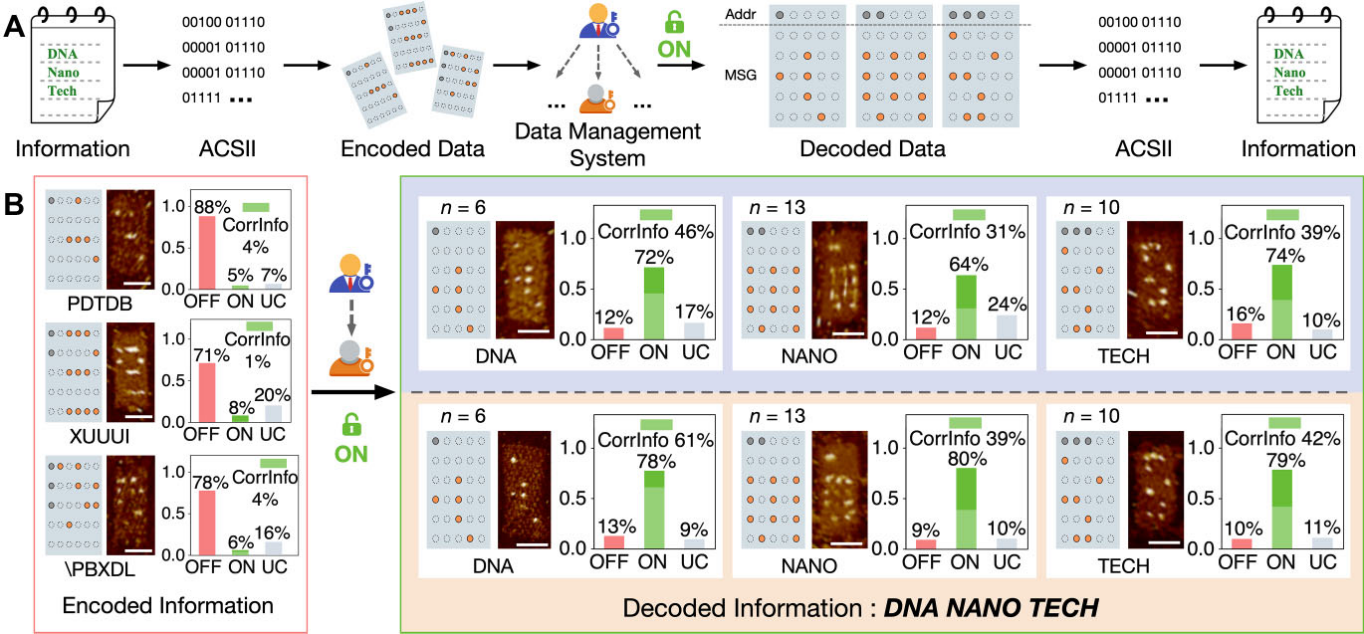
Accessing encoded data using user and admin keys. (**A**) Workflow of data access using DDC structure. The information is first converted into binary strings using ASCII. Then, the binary data are arranged and stored in the 5 × 5 array on the DDC, with an address row added above the data array. The ciphertext is accessed using either the admin or user key. The decoded data are arranged into ordered binary digits according to the address bits and then converted back to plaintext. (**B**) Data access using user and admin keys. In the OFF conformation, the message reads “PDTDB XUUUI \PBXDL” (red box, left). The numbers of quantified DDC structures are 203, 167, and 310, respectively. In the ON conformation, the message reads “DNA NANO TECH” (green box, right). For user access, the numbers of quantified DDC structures are 116, 319, and 178, respectively; for admin access, 264, 193, and 293 structures are counted. OFF, DDC with OFF conformation; ON, DDC with ON conformation; UC, uncertain conformation; CorrInfo, correct information. Vertical axis, fraction of the target conformation or pattern. Scale bars, 50 nm.

Based on the reconfigurable DDC, we developed a data encoding system where the information was stored on the nanostructure and accessed using admin and user keys. Figure 3A illustrates the workflow of this system. First, the information was converted into binary digit strings using ASCII. The binary message was then arranged and stored in the 5 × 5 array on the DDC (Supplementary Fig. S15). In this array, an STV-attached bit was marked as “1”, while an unattached bit was marked as “0”. An additional address row was incorporated above the message array. Prior to access, the address bits were mixed with the message bits, resulting in a disordered data strings that enhanced information concealment. Decoding and access to the ciphertext were achieved by transforming the DDC conformation using either the user or admin key. Once activated, the data were rearranged into ordered binary digits according to the address bits and subsequently converted back to original information using ASCII.

To assess the feasibility of the system, we encoded a phrase onto the DDC structure (Fig. 3B). The phrase and address were initially encrypted as disordered ciphertext: “PDTDB XUUUI \PBXDL”. When accessed using the admin or user keys, the DDC with ON conformation revealed the ordered and correct information (CorrInfo): “DNA NANO TECH”. AFM analysis showed that prior to the addition of keys, only a small percentage of DDC structures (4%, 1%, and 4%, respectively) displayed the correct information for the three words (Supplementary Figs S16–S18). After the addition of the admin key, the percentage of nanostructures showing the correct information increased significantly to 46%, 31%, and 39%, respectively. Samples treated with the user key demonstrated CorrInfo yields of 61%, 39%, and 42%, respectively (Tables S4–S6).

It is worth noting that the yield of CorrInfo depends not only on the efficiency of DDC transformation but also on the efficiency of STV binding. For the binary bits array on the original DDC structure, the “0” dot is made up of a regular staple strand, while the “1” dot is made up of a biotin-modified staple. To obtain the correct information, the DDC must transform to the ON conformation and all biotin-modified staples must successfully bind to STV. When an excess of STV is added, it is assumed that the binding of STV to each biotin site is independent. Therefore, the theoretical yield of CorrInfo, ${{y}_{{\rm theo}}}$, can be calculated using ,Eq. (2):


(2) \begin{eqnarray*}
{{y}_{{\rm theo}}} = p \times {{q}^n},
\end{eqnarray*}

where $p$ represents the estimated transformation ratio of the DDC structure (89% for the user key and 76% for the admin key, Fig. 2C), $q$ denotes the percentage of STV correctly bound to biotin-modified staple strands (∼95%) [13], and $n$ indicates the number of available biotin sites in the data and address bit array. As the value of $n$ increases, the theoretical yield of CorrInfo decreases. This theoretical prediction is supported by experimental results. The “DNA” pattern, with the fewest bit dots, showed the highest yield, while the “NANO” pattern, with the most dots, exhibited the lowest yield (in both the user key and the admin key experiments).

A special design was employed to ensure the accuracy of information yielded from STV–biotin conjugation. Each STV molecule contains four high-affinity biotin-binding sites, which poses the risk of adjacent biotin-modified staples within the data array binding to the same STV molecule. This could lead to uneven linkage and incorrect data interpretation. To mitigate this risk, we selectively introduced blank columns within the data array of plaintext words. For instance, in the array of “DNA,” we added a blank column between the bit columns of “D” and “N”. These blank columns are confined within the bit array of the word and do not interfere with the reading and interpretation of phrases. Additionally, an excess amount of STV relative to biotin sites was used during the experiments to further reduce the likelihood of a single STV molecule binding to multiple biotin sites.

### Hierarchical access to encoded data in multi-user scenario

**Figure 4.**
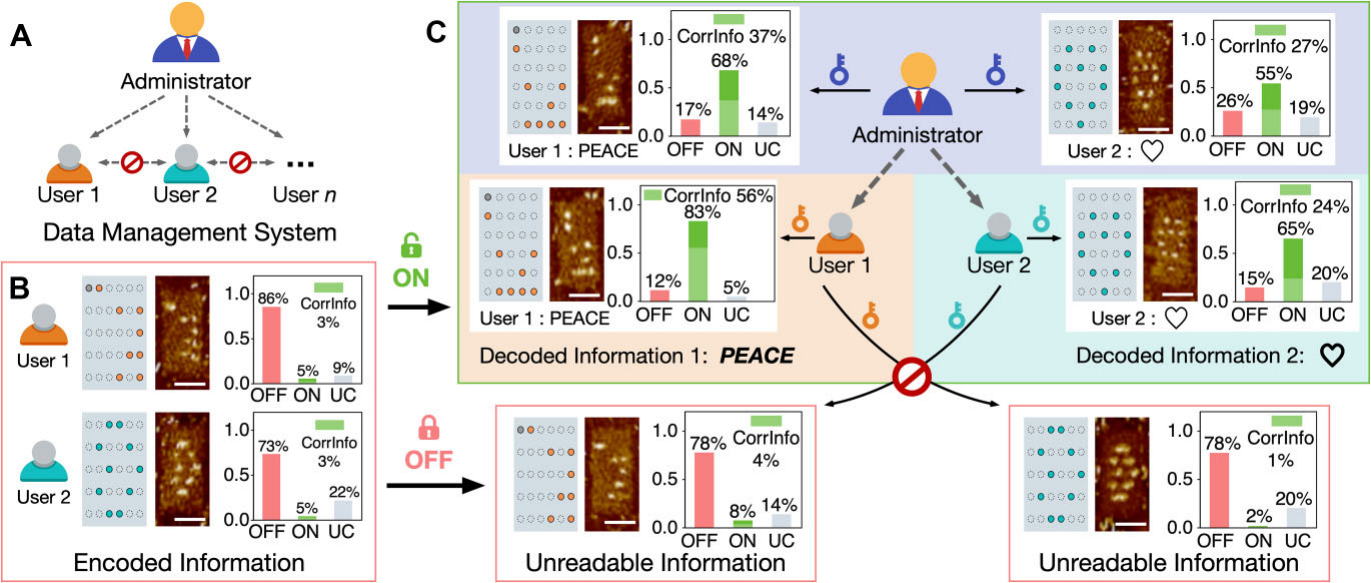
Data management system: accessing encoded data using hierarchical keys with differential permission. (**A**) Schematic representation of the hierarchy relationship between the admin and users. (B, C) Implementation of the multi-user data management system. (**B**) Distinct DDC structures are used to store data for different users. A total of 257 and 222 DDC structures are quantified from AFM images for the samples corresponding to user 1 and user 2, respectively. (**C**) The admin key enables access to data from both users (top row of green box), with 132 and 139 structures counted for user 1 and user 2, respectively. In contrast, user keys allow access only to their own data (bottom row of green box), with 180 structures for user 1 and 219 for user 2. When user keys are used to access unauthorized data (red boxes), 191 and 205 structures are observed for the unreadable data of user 1 and user 2, respectively. Vertical axis, fraction of the target conformation or pattern. Scale bars of AFM image, 50 nm.

Building on the successful use of both the admin and user keys to access encoded data, we expanded our data management system to accommodate a multi-user scenario using the DDC structure. This hierarchical access system provided differentiated permissions for administrators and individual users (Fig. 4A). The administrator was granted full access to all users’ data, while each user was restricted to accessing only their own data. This system offered an approach to secure data management by protecting user privacy and limiting access to authorized personnel only.

To store multi-user data, we designed a new DDC structure (DDC-2; Supplementary Fig. S19). DDC-2 utilized the same scaffold (M13mp18) as user 1’s nanostructure carrier (DDC-1; Supplementary Fig. S1) but had a different collection of staple sequences. While the overall shape of DDC-2 was identical to that of DDC-1, the sequences of its gap regions and edge staples differed, ensuring that user 2’s key was orthogonal to user 1’s key. We encoded the word “PEACE” on DDC-1 using ASCII encoding and assigned the address bits. To illustrate data diversity, we encoded and stored a patterned dataset in the form of a heart-shaped icon on DDC-2 (Fig. 4B).

Hierarchical access was established based on the distinct properties of the biochemical reactions triggered by the admin and user keys. The admin key (Klenow polymerase) facilitated the DDC transformation through nonspecific gap filling and PTSD reactions, allowing a “one-to-many” relationship for accessing data associated with multiple users. The user key (DNA strand set) induced structural changes via specific TMSD reactions, establishing a “one-to-one” relationship with user data, thus limiting access to only the corresponding user’s data. Statistical analysis of the AFM images confirmed the hierarchical relationship between the administrator and the users (Fig. 4C and Supplementary Figs S20–S23). The administrator had permission to access the data of user 1 and user 2 with CorrInfo yields of 37% and 27%, respectively. When user 1 and user 2 accessed their own data using corresponding keys, the CorrInfo yields were 56% and 24%, respectively (Supplementary Fig. S24). However, when users attempted to access data with mismatched keys, the CorrInfo yields dropped to 4% and 1%, similar to the values observed in encoded samples (Supplementary Tables S7 and S8). Although the CorrInfo yields for user 2, obtained using both the admin key and user 2’s key, were lower than ideal, they were significantly higher than the yields obtained with a mismatched user key. Thus, we concluded that the admin key and the matched key could effectively access to user 2’s data.

## Discussion

In summary, we developed a DNA-based data management system that enabled hierarchical access to encoded information using both admin and user keys. ASCII-formatted data were stored on a reconfigurable DDC structure through pre-arranged STV binding sites. Retrieval of the encoded information was achieved by inducing conformational changes of the DDC structure through either TMSD or PTSD reactions. Incorporating edge strand displacement via TMSD or PTSD reactions enhanced the efficiency of DDC conformational changes (Supplementary Fig. S4 and Supplementary Table S9). The user key was represented by the invading strands in the TMSD reaction, while the polymerase in the PTSD reaction functioned as the admin key. We demonstrated that both admin and user keys could successfully access the identical encoded message. In a multi-user situation, the admin key provided universal access to all users’ data, whereas the user keys granted access only to their respective data. This system offered an approach to secure data management by protecting user privacy and limiting access to authorized personnel only.

Several potential efforts could further improve the data management system. The number of users in the system is constrained by the length and sequence diversity of the scaffold strand. In the design of DDC-2, the single-stranded regions of the scaffold strand on the sides were modified by moving the breakpoint of the scaffold sequence, thereby generating a distinct triggering strand set for user 2’s key, different from user 1’s key. Theoretically, this method could produce more than 7000 unique DNA user keys. However, ensuring the orthogonality of trigger strands requires a great range of scaffold breakpoint shifts, which limits the total number of user keys. A promising solution to this limitation is the synthesis of custom, longer scaffold strands, which would allow for the generation of a larger pool of orthogonal trigger strands and accommodate more users. Additionally, the spatial arrangement of STV sites significantly affects the accuracy of data readout (Supplementary Fig. S25). Expanding scaffold length and designing larger carrier structures could not only increase the capacity for orthogonal key generation but also enhance the precision of data retrieval.

A small number of DDC structures in the ON conformation (false positives) can appear even in the absence of a trigger (Supplementary Fig. S16). To further reduce the occurrence of false positives, one potential strategy is to optimize the base sequences at the crossover points of the anti-junctions within the DNA arrays. As discussed in the original work, the overall conformation and transformation efficiency of the domino DNA array are highly influenced by the base sequences at the crossover points of anti-junctions. By carefully selecting the scaffold breakpoint position in caDNAno during the design phase, one can bias the structure toward adopting the OFF conformation. This design-based approach offers a practical method to further minimize false activation and enhance encryption reliability when the trigger is absent.

Improving information capacity and security is also essential. One effective approach is to prepare mixed samples containing structures that store different messages (Supplementary Fig. S26). By integrating multiple distinct DDCs into a single sample, the information capacity per AFM characterization increases directly. Furthermore, the number of possible data patterns in such mixed samples also grows exponentially, significantly enhancing the overall security of the system.

The rapid advancements in DNA information storage technology are creating new demands for functions such as data management and access control. In the future, DNA-based storage and management systems may require the integration of diverse biomolecules and biochemical reactions to perform a wide range of functions comparable to those of traditional silicon-based systems. The proposed data management system provides a demonstration of hierarchical data access with the help of DNA polymerase. We envision that this demo could inspire the development of more sophisticated information management functions to improve DNA information storage technology.

## Supplementary Material

gkaf835_Supplemental_Files

## Data Availability

The data underlying this article will be shared on reasonable request to the corresponding author.
