# Supplementary material for: Hierarchical access to encoded data on DNA nanostructures using administrator and user keys
Source: Nucleic Acids Res. 2025 Aug 27;53(16):gkaf835. doi: 10.1093/nar/gkaf835 (PMC12390752; doi:10.1093/nar/gkaf835)
Supplement: gkaf835_Supplemental_Files [file gkaf835_supplemental_files.zip › R2-Clean-Supplementary file-20250725.pdf]

Supporting Information for

Hierarchical Access to Encoded Data on DNA Nanostructures Using Administrator and User Keys

AUTHORS

Kuiting Chen[1], Sisi Fan[2,3], Na Liu[2,3], Jie Song[4,*], Linqiang Pan[1,*]

[1] Key Laboratory of Image Information Processing and Intelligent Control of Education Ministry of China, School of Artificial Intelligence and Automation, Huazhong University of Science and Technology, Wuhan, Hubei, 430074, China
[2] 2nd Physics Institute, University of Stuttgart, Stuttgart, 70569, Germany
[3] Max Planck Institute for Solid State Research, Stuttgart, 70569, Germany
[4] Hangzhou Institute of Medicine, Chinese Academy of Sciences, Hangzhou, Zhejiang, 310022, China

* To whom correspondence should be addressed. Email: lqpan@mail.hust.edu.cn.
Correspondence may also be addressed to Jie Song. Email: songjie@him.cas.cn

CONTENT

1. Supporting Figures

2. Supporting Tables

References

1. Supporting Figures

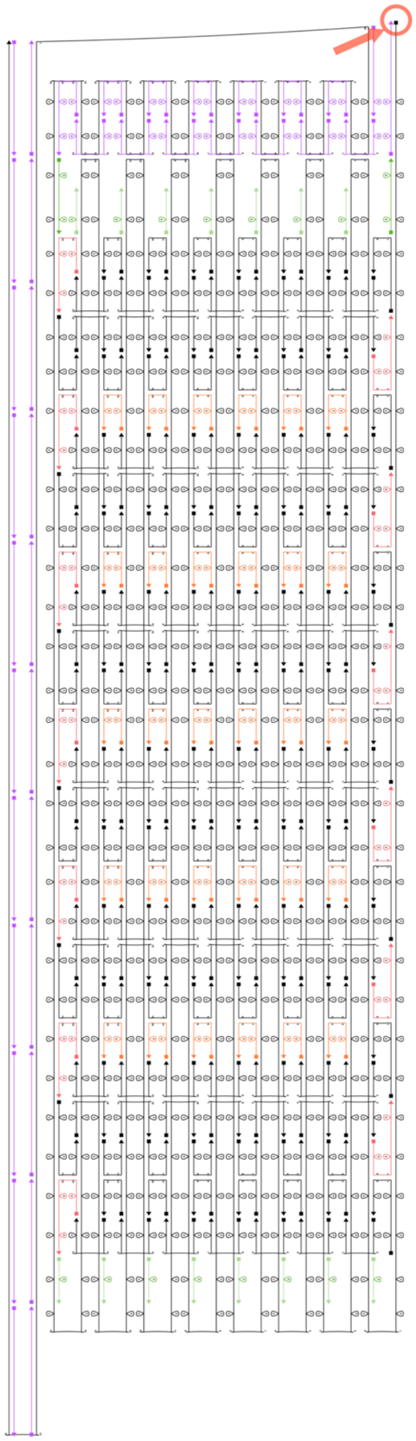

Figure S1: Layout of DDC-1 (1). Black line: scaffold (M13mp18); grey line: common staples; green line: primers; red line: edge strands, orange strands: data array. Purple strands hybridized with the redundant fraction of the scaffold strand to form a double-stranded loop, avoiding the risk of undesired pairing of the redundant scaffold with input DNA strands. The red circles and arrow indicate the breakpoint of scaffold strand.

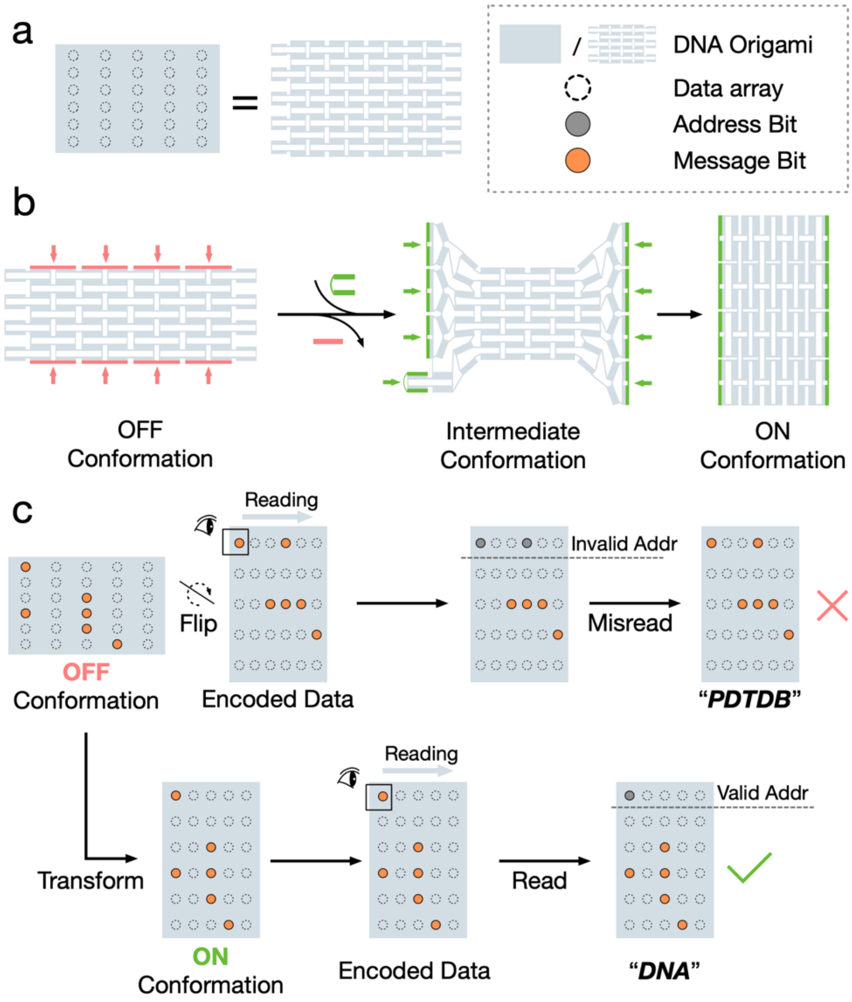

Figure S2: (a) Diagram of nanostructure acting as the DNA-based carrier of encoded data. It is worth noting that although we used orange dots and grey dots to distinguish between information and address bits in the schematic, both types of dots were marked with biotin–STV conjugation. (b) Diagram of reconfiguring DDC. The general shapes of DDC are largely same before and after the transformation (two-dimensional rectangle). (c) Diagram of accessing to the patterned data according to the reconfiguring DDC. When accessing the user's information, the short sides of DDC were located on the top and bottom. The address bits were located in the upper left corner of DDC. The information should be read from left to right. Following these rules, only when DDC transformed conformation, could the observer obtain correct readout, including the address and user's message.

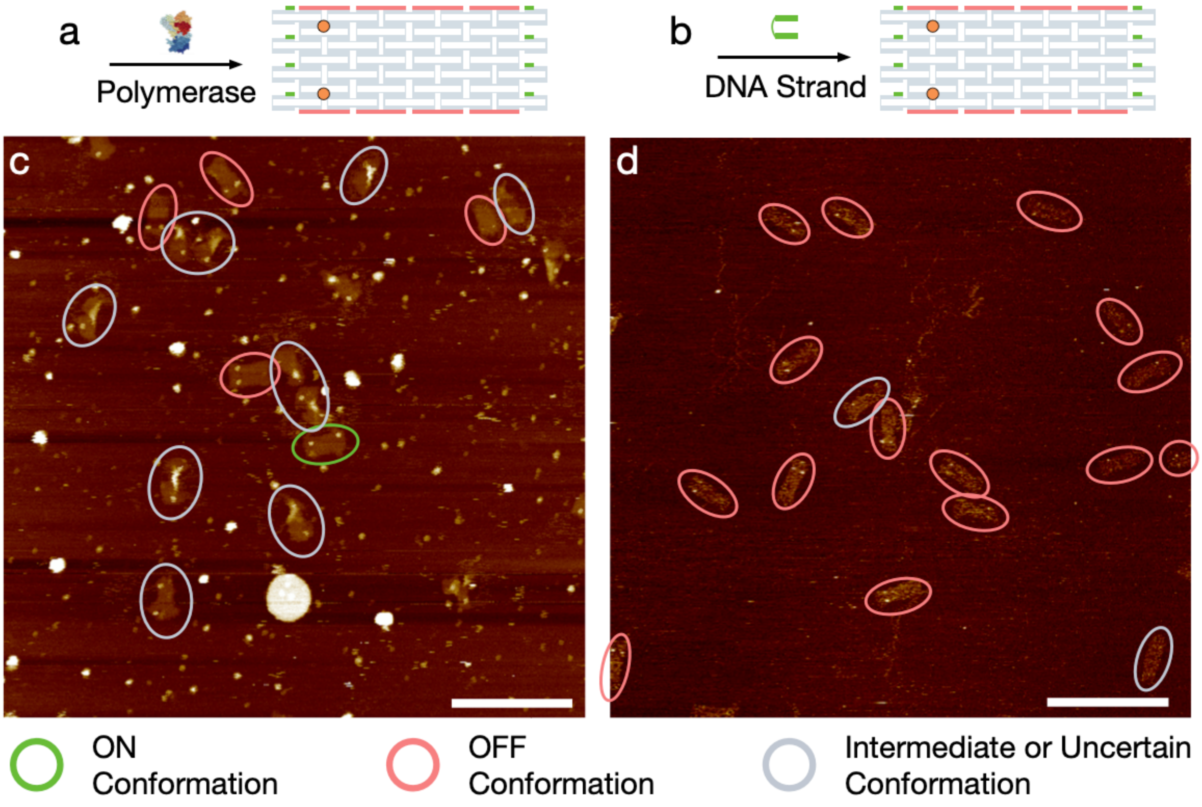

Figure S3: Failed transformation of the DDC structure with full edge staples to the ON conformation. (a & b) Schematic representation of the transformation of the DDC structure using polymerase (a) and DNA strand set (b). (c & d) AFM image of the failed-transformed DDC structure using polymerase (c) and DNA strand set (d). Scale bar, 400 nm.

We observed a different transformation efficiency compared to previous studies using domino DNA arrays with different scaffold sequences (1). The overall conformation and transformation efficiency of the domino DNA array are highly influenced by the base sequences at the crossover points of anti-junctions (2). In particular, the four base pairs at each four-way junction play a critical role in determining the structural assembly result. Therefore, even small changes to the scaffold sequence—such as switching from M13mp18 to p7560 or altering the breakpoint position—can lead to global shifts in the base composition at these crossover sites. These sequence changes propagate throughout the structure and can significantly affect the transformation efficiency of the array.

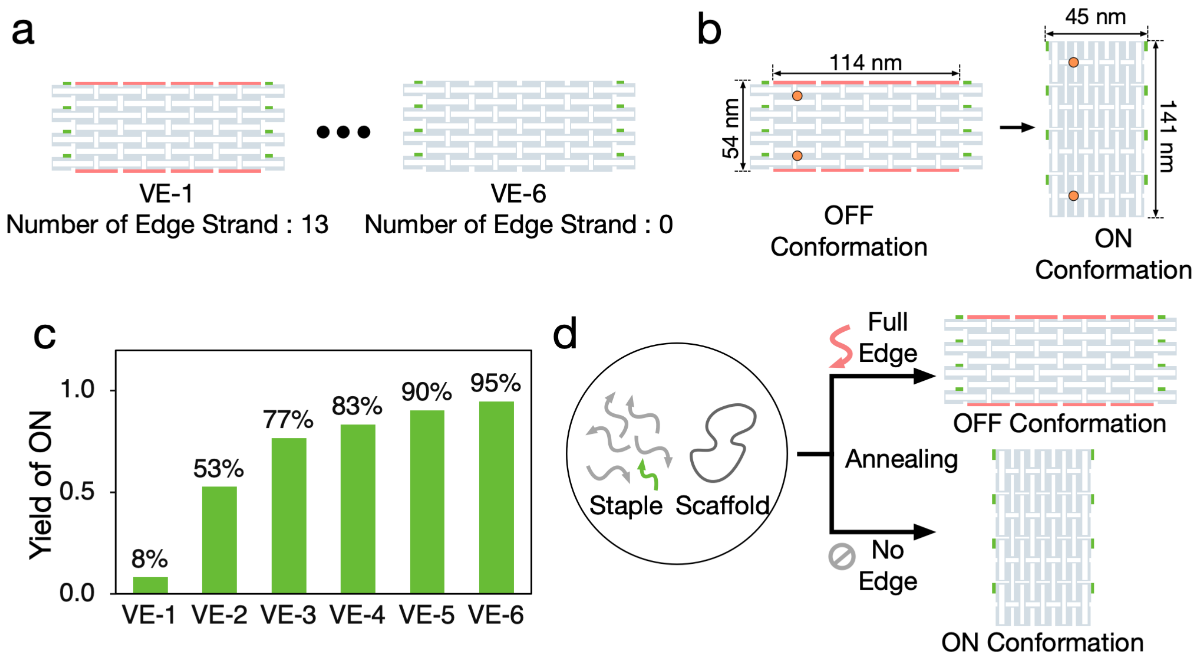

Figure S4: Relationship between the number of edge strands and the conformation of DDC. (a) Six DDC variants with varying numbers of edge strands. The number of edge strands for the six variants, designated VE-1 through VE-6, is 13, 11, 8, 6, 4, and 0, respectively. See Figure S5-S10 for detailed diagram. (b) Size of the DDC structure. The dimensions of DDC show little difference before and after transformation. Hence, it is necessary to use STV (orange dots) to indicate the transformation results. (c) Histogram of the percentage of ON conformations in the variant samples. Six variants yield 8%, 53%, 77%, 83%, 90%, and 95% of the ON conformation, respectively. The statistics of the AFM images (Figures S5-S10) indicated that the DDC structures produced by one-pot annealing tended to exhibit ON conformation when the number of edge staples decreased. Meanwhile, the DDC structure without edge strands had the highest percentage of ON conformations. (d) Schematic representation of experimental results with varying numbers of edge strands. DDC structures with full edge strands predominantly exhibited the OFF conformation after one-pot annealing. In contrast, DDC structures without edge strands showed the highest percentage of ON conformation.

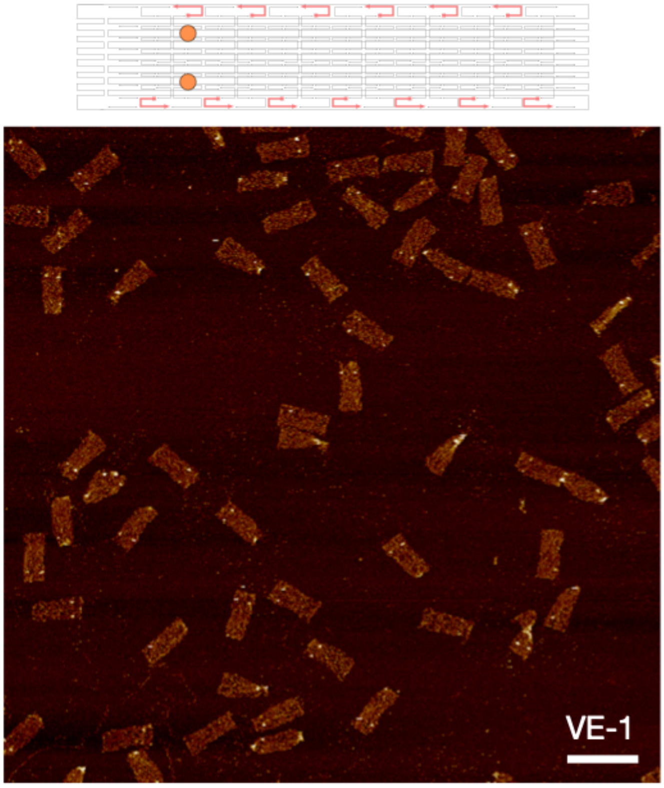

Figure S5: Diagram and representative AFM image of DDC with 13 edge strands (VE-1). Two STV molecules adhered to the short side of the OFF conformation and presented on the long side of the ON conformation. Scale bars, 200 nm.

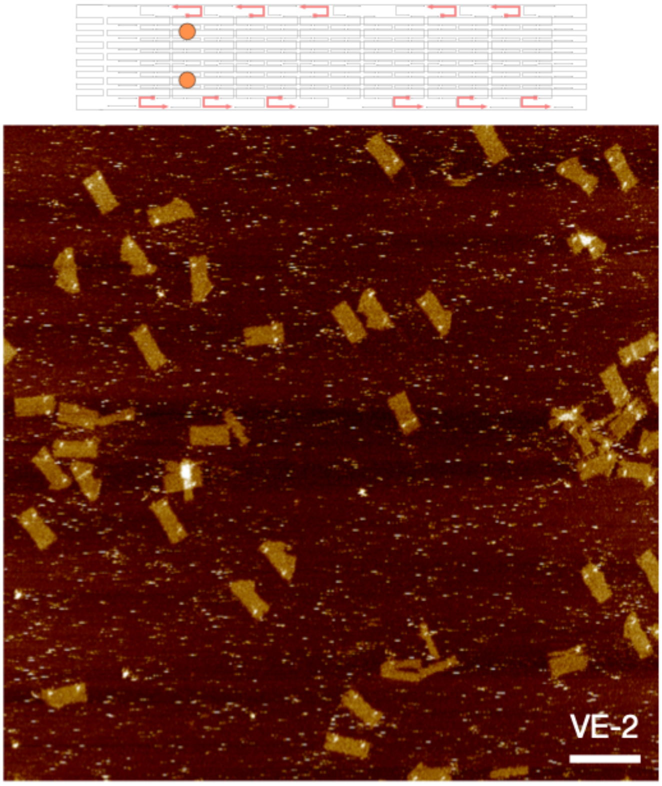

Figure S6: Diagram and representative AFM image of DDC with 11 edge strands (VE-2). Scale bars, 200 nm.

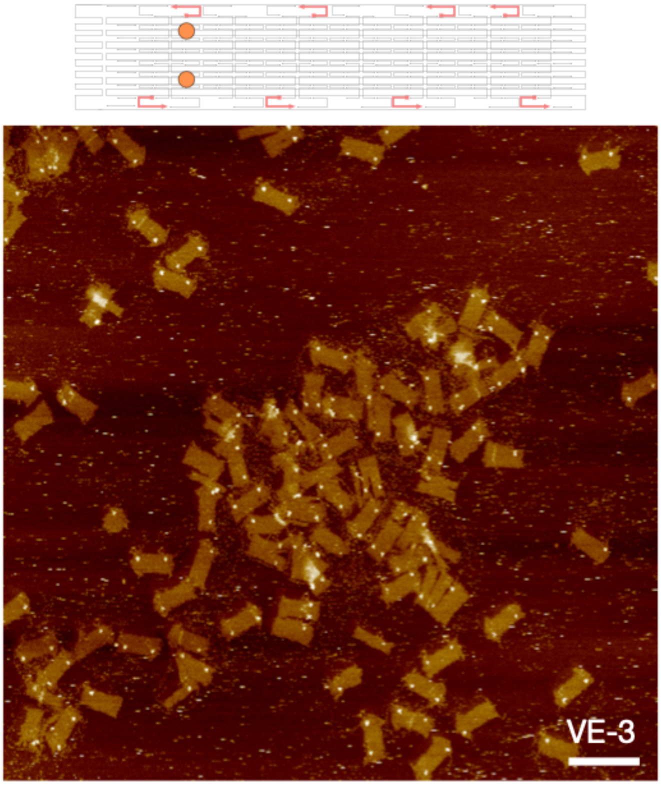

Figure S7: Diagram and representative AFM image of DDC with 8 edge strands (VE-3). Scale bars, 200 nm.

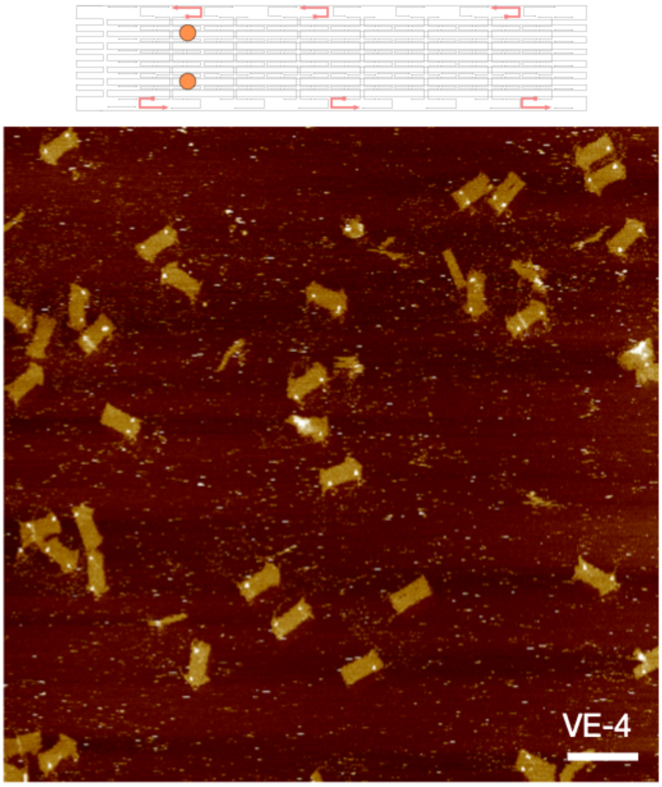

Figure S8: Diagram and representative AFM image of DDC with 6 edge strands (VE-4). Scale bars, 200 nm.

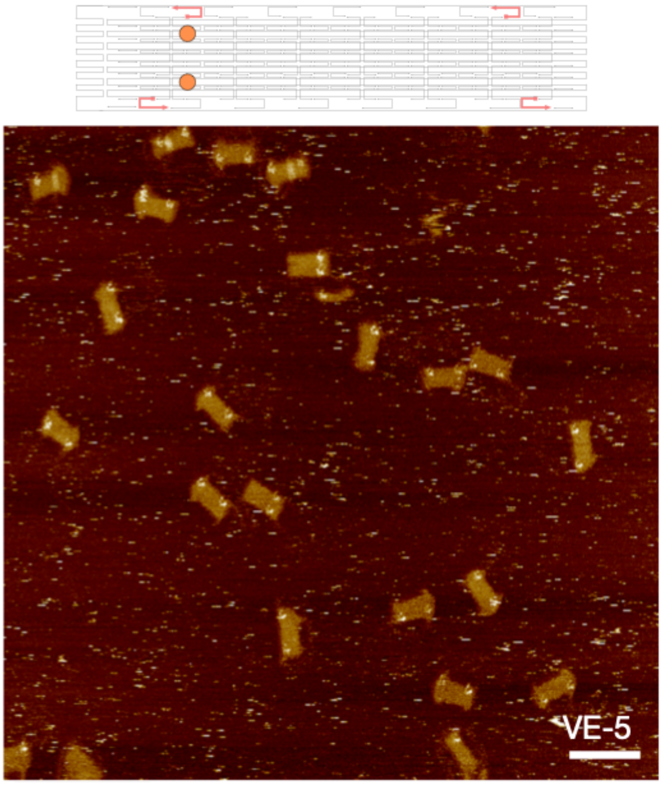

Figure S9: Diagram and representative AFM image of DDC with 4 edge strands (VE-5). Scale bars, 200 nm.

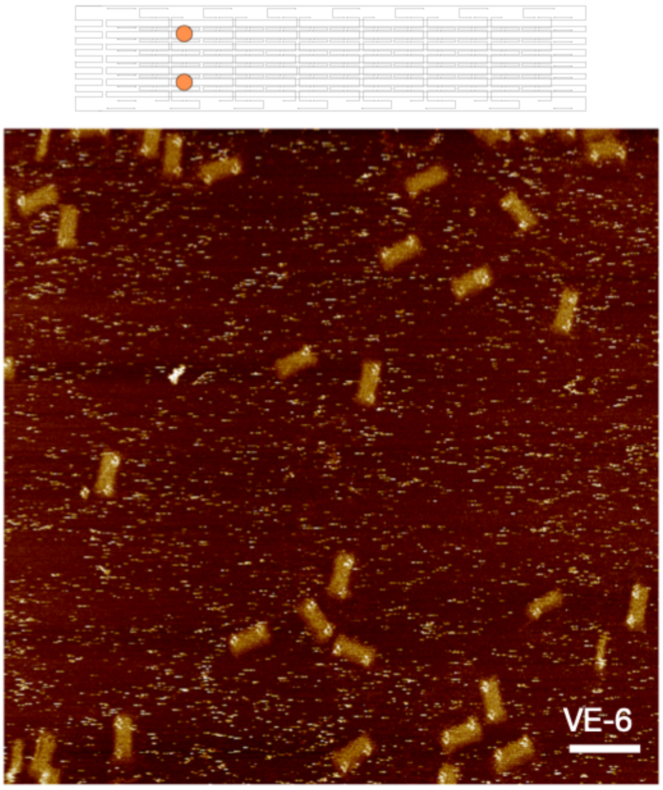

Figure S10: Diagram and representative AFM image of DDC with no edge strands (VE-6). Scale bars, 200 nm.

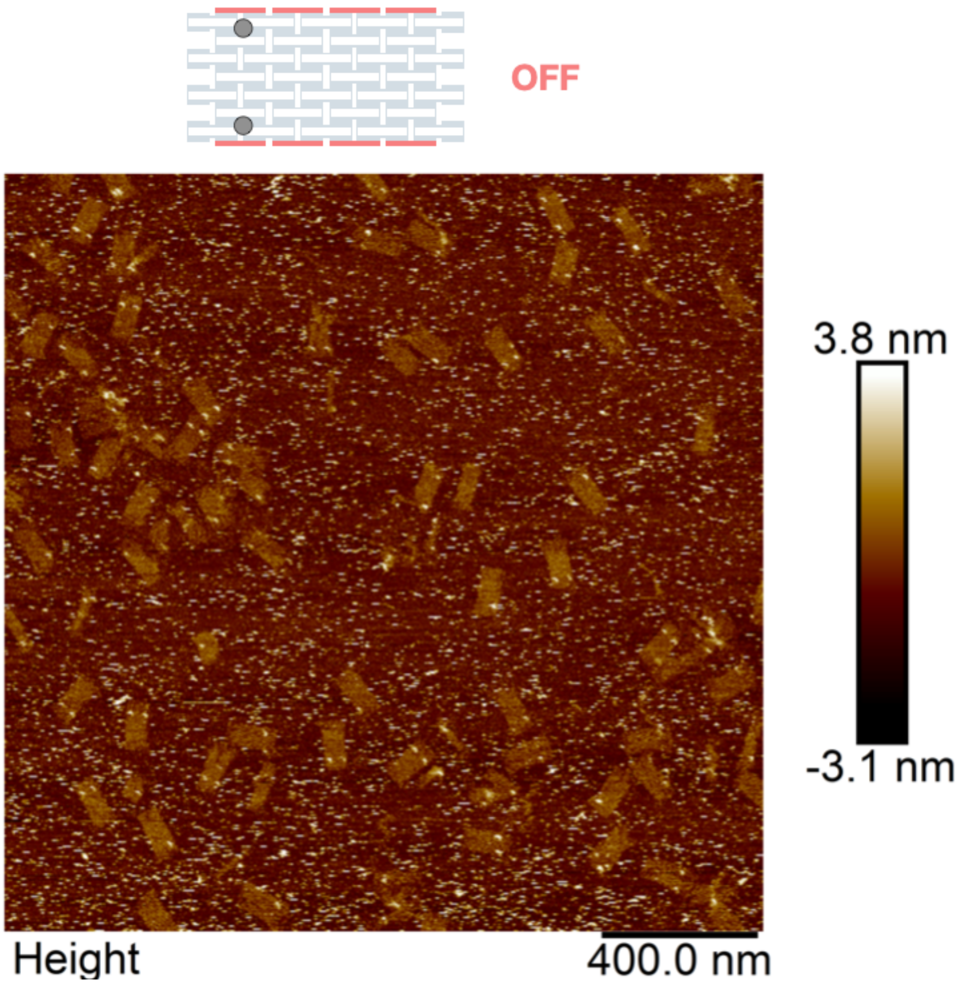

Figure S11: Representative AFM image of two-STV labeled DDC structure before the transformation.

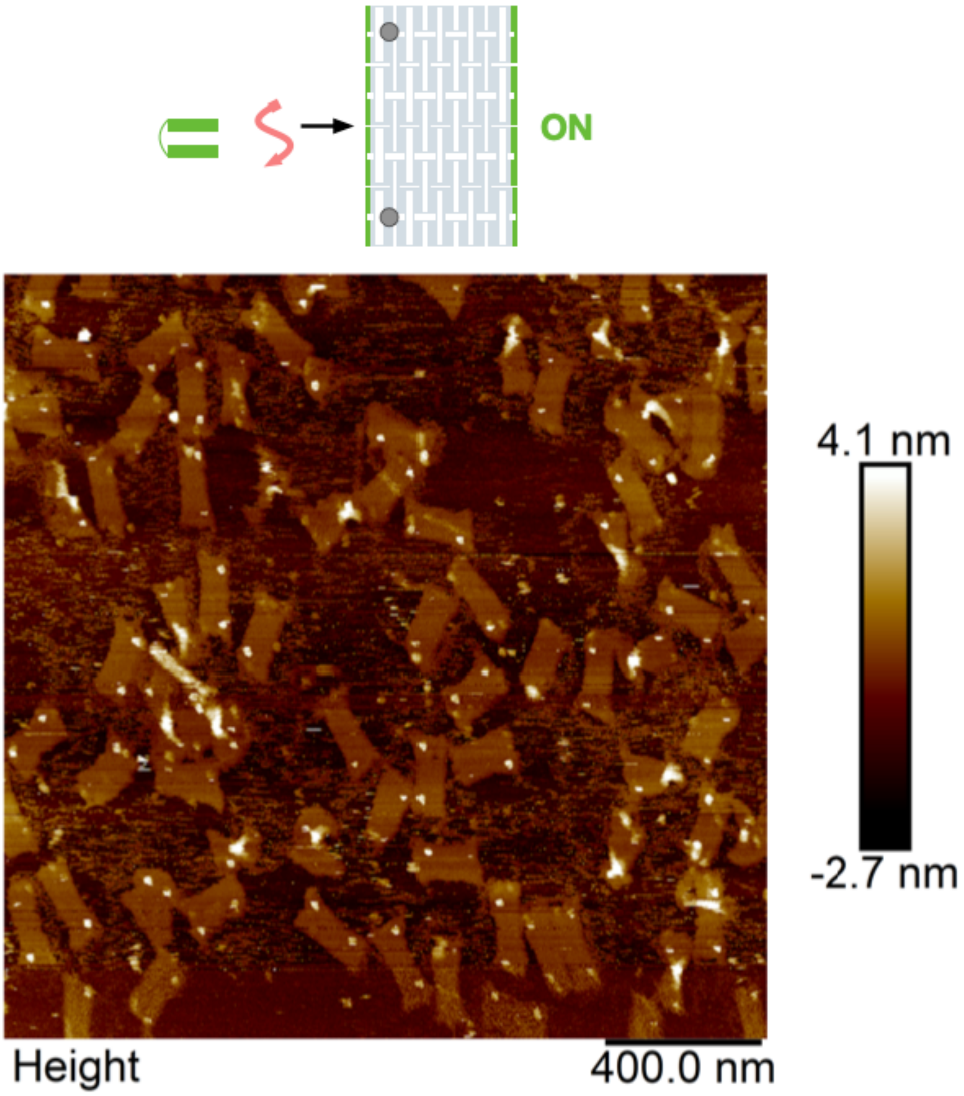

Figure S12: Representative AFM image of two-STV labeled DDC structure after the transformation using the DNA strand set.

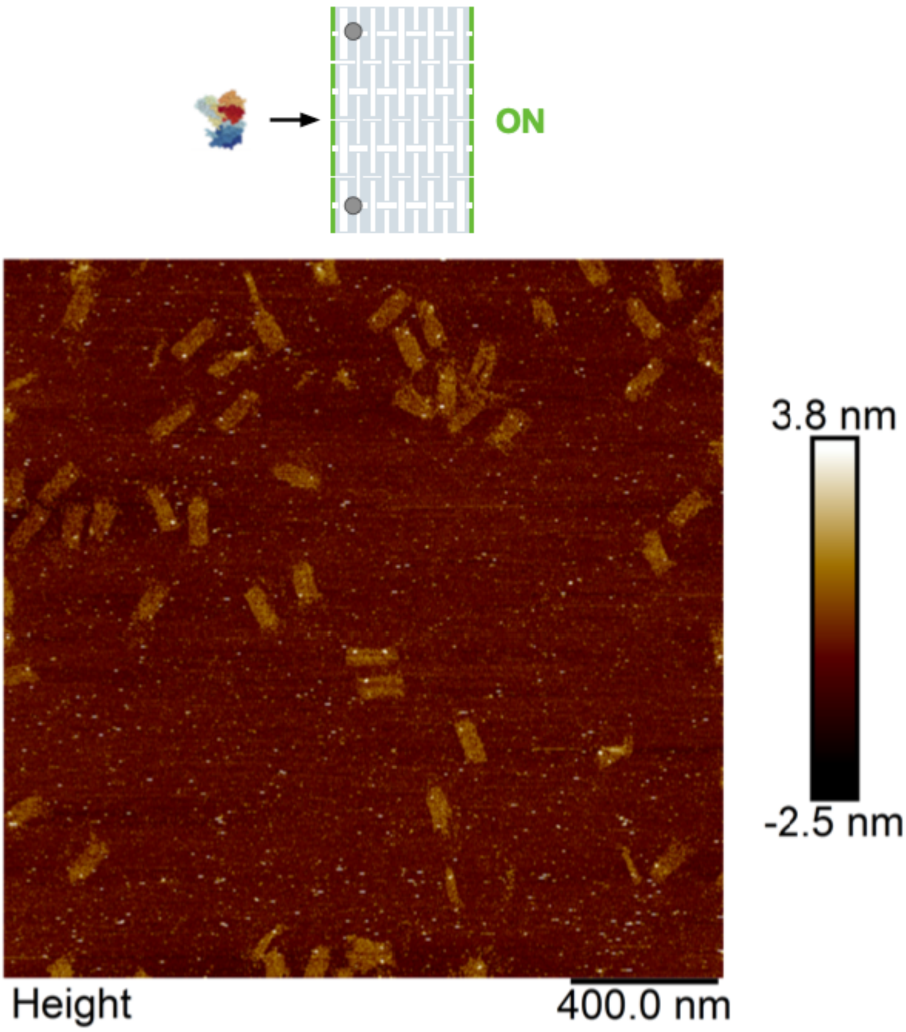

Figure S13: Representative AFM image of two-STV labeled DDC structure after the transformation using polymerase.

In the polymerase-assisted gap filling and PTSD reactions, the strand displacement activity of Klenow may disrupt the binding between common staples and the DDC structure. We have discussed this issue in our previous study on DNA kirigami (3, 4). The experience indicates that the inner staples are less likely to be removed unexpectedly by Klenow. Therefore, reducing the concentration and duration of Klenow can mitigate the undesired removal of internal common staples in the DDC structure to some extent. In AFM images, the nanostructures with ON conformation transformed by polymerase, especially at the edges and sides, show some damage, indicating that it is challenging to completely avoid Klenow-induced disruption of common staples. However, since the staples constituting the data pattern are located in the interior of the structure, Klenow's impact on these staples is limited, thereby scarcely affecting the application of the DDC structure in data encoding.

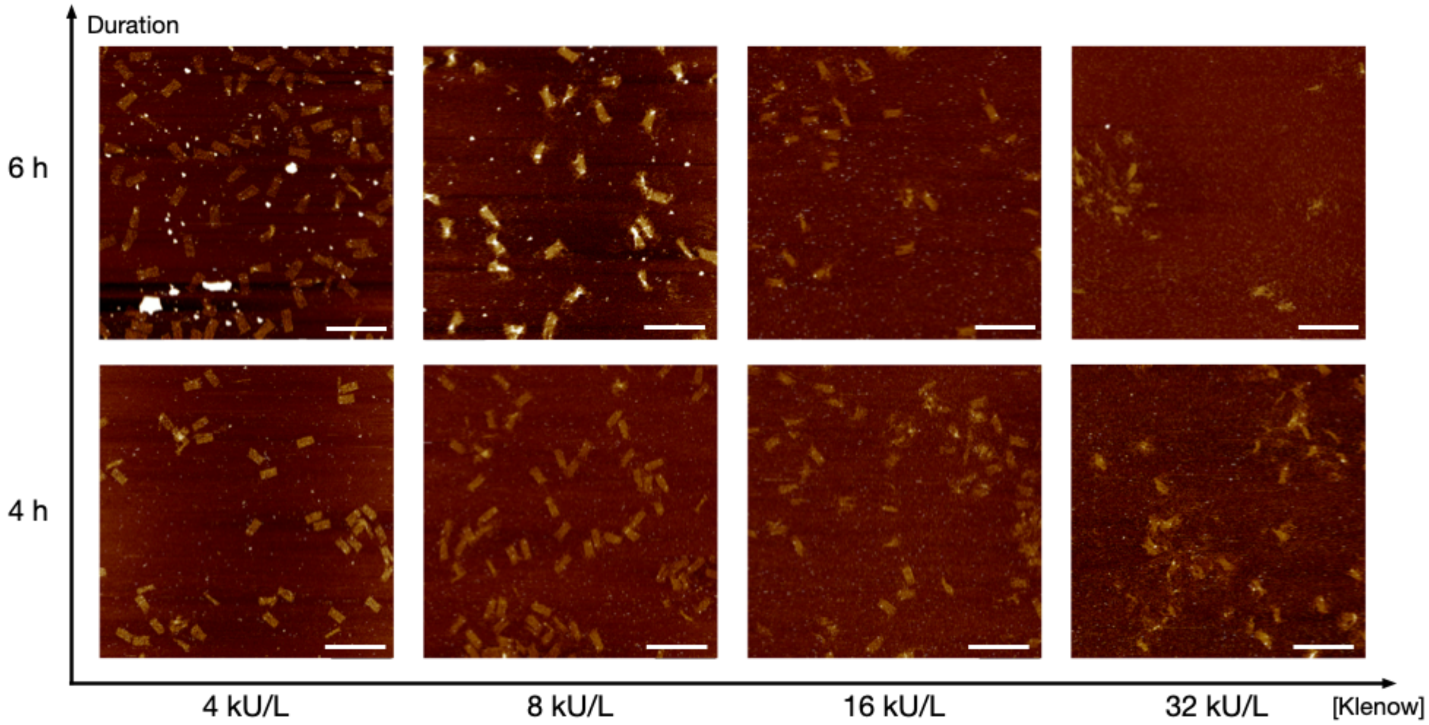

Figure S14: AFM images of the two-STV labeled DDC structure after polymerase treatment. An orthogonal test was performed to evaluate the effects of two parameters: polymerase concentration and treatment duration. Scale bars, 400 nm.

When the reaction duration reached 6 hours, the DDC structures in the samples exhibited varying degrees of damage. Notably, in the sample treated with 32 kU/L polymerase, barely any intact carrier structures were observed. After 4 hours of reaction time, significant structural damage to the DDC was evident in samples treated with polymerase concentrations of 16 kU/L and 32 kU/L. Conversely, samples treated with 4 kU/L polymerase showed intact structures but exhibited limited transformation of the DDC conformation. Among the eight tested conditions, the highest percentage of successfully transformed DDC structures was observed only in the sample treated with 8 kU/L polymerase for 4 hours. These results indicate that polymerase-triggered DDC transformation requires precisely controlled reaction conditions.

The final concentration of DNA origami in all eight samples was approximately 12.5 nM. As the reaction substrate, the origami concentration influenced the optimal polymerization reaction parameters, suggesting that any changes in substrate concentration would

necessitate adjustments in reaction conditions. Stringent experimental protocols and the need for precisely tuned reaction parameters ensure the security of the admin key during polymerase-driven DDC transformation and access to encoded data.

ASCII	Letter	Pattern	ASCII	Letter	Pattern	ASCII	Letter	Pattern
0100 0001	A	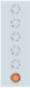	0100 1010	J	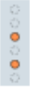	0101 0011	S	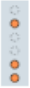
0100 0010	B	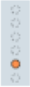	0100 1011	K	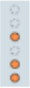	0101 0100	T	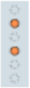
0100 0011	C	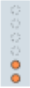	0100 1100	L	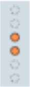	0101 0101	U	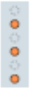
0100 0100	D	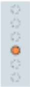	0100 1101	M	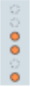	0101 0110	V	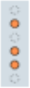
0100 0101	E	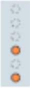	0100 1110	N	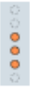	0101 0111	W	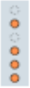
0100 0110	F	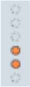	0100 1111	O	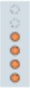	0101 1000	X	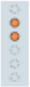
0100 0111	G	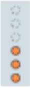	0101 0000	P	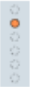	0101 1001	Y	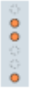
0100 1000	H	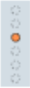	0101 0001	Q	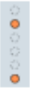	0101 1010	Z	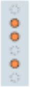
0100 1001	I	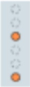	0101 0010	R	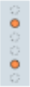			

Figure S15: Comparison chart between ASCII codes of letters and patterns on the data array. In the 8-bit binary ASCII code, the first three bits (010) of each letter are identical. Therefore, only the last five bits were arranged on the DDC.

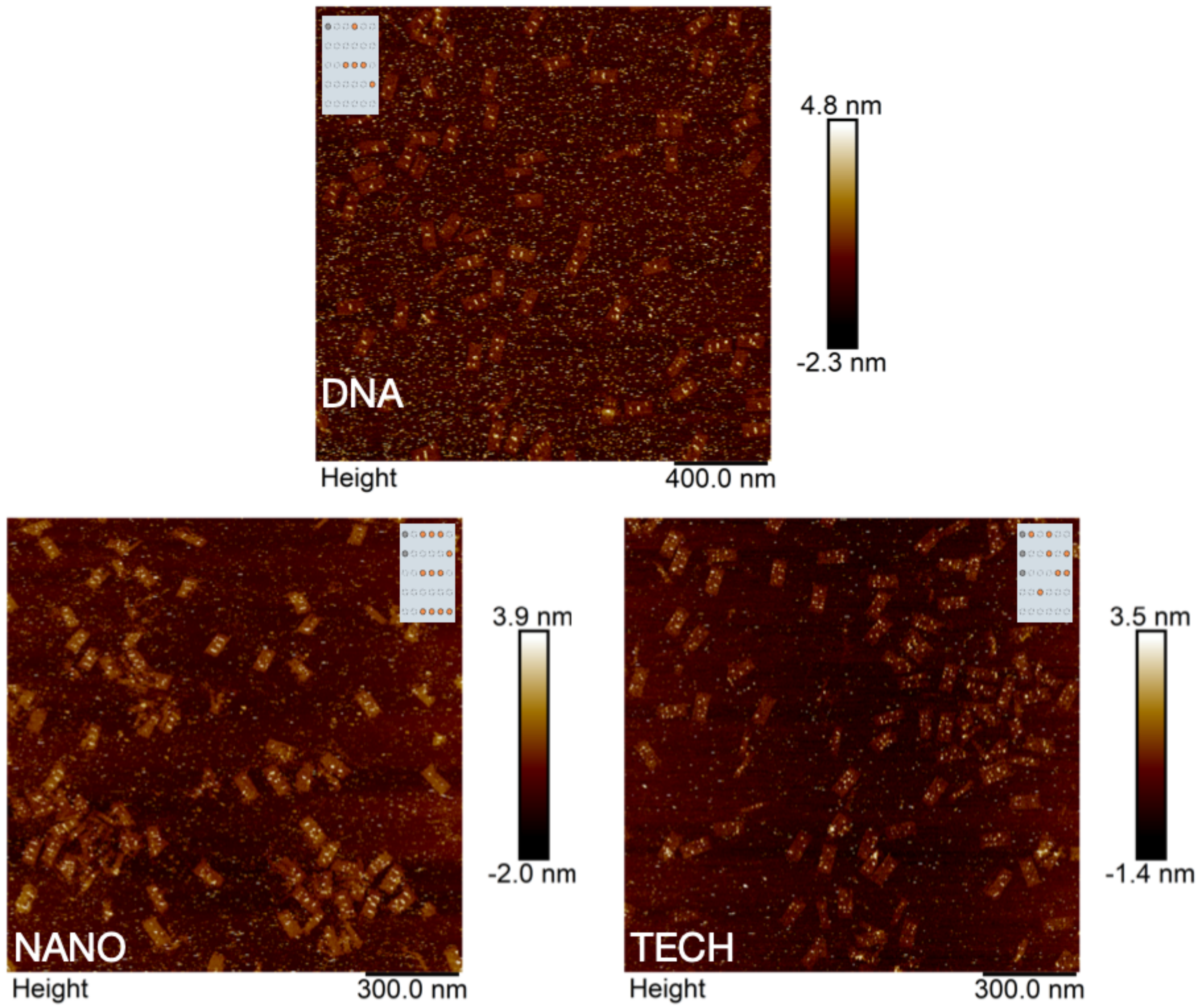

Figure S16: Representative AFM images of DDC structure with ciphertext.

A small number of DDC structures in the ON conformation (false positives) can appear even in the absence of a trigger. However, this does not result in significant information leakage. While there is a certain probability that STV molecules bind correctly to these structures, the likelihood of a false positive both forming and presenting coherent, readable information is extremely low (left columns of Figure 3b and Figure 4b).

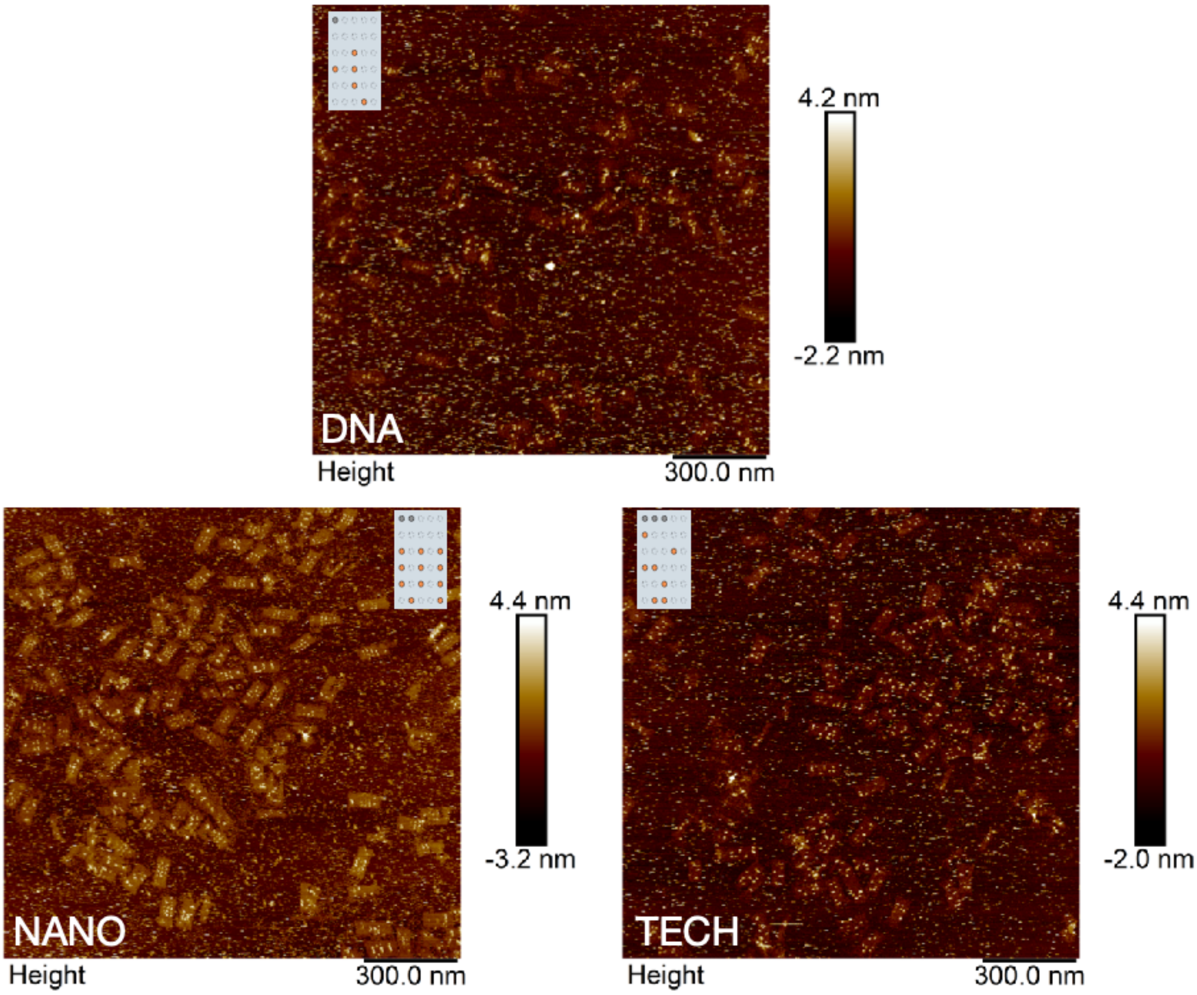

Figure S17: Representative AFM images of DDC structure with plaintext “DNA NANO TECH” decoded by the admin key.

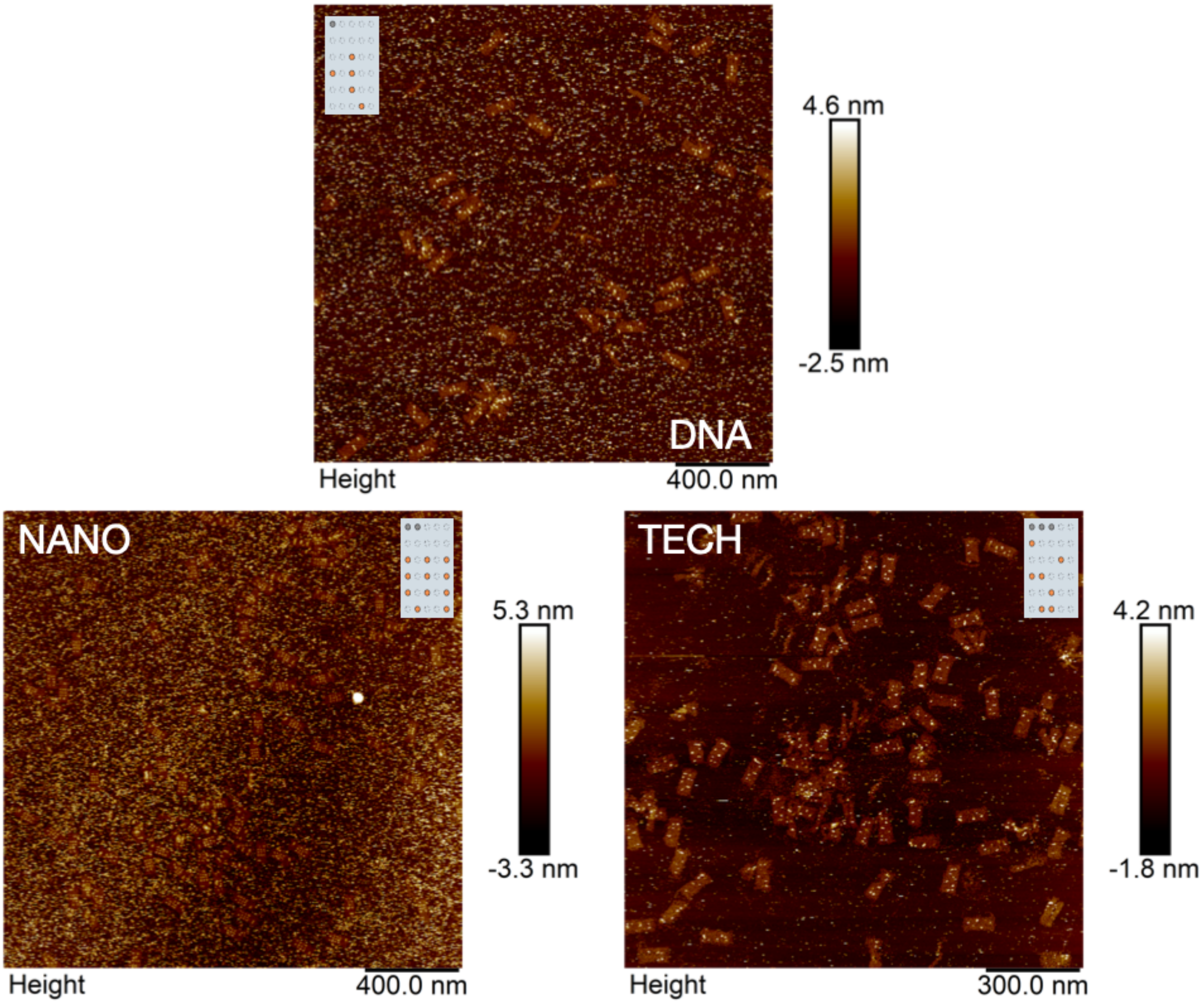

Figure S18: Representative AFM images of DDC structure with plaintext "DNA NANO TECH" decoded by the user key.

In AFM images, some structures are classified as uncertain structures—a category of DNA origami in which the state, conformation, or encoded information cannot be reliably determined due to missing STV spots on the surface. Structures lacking two, three, or more STVs fall into this category. In such cases, the proportion of any particular STV pattern is typically very low, making it difficult to extract meaningful information from these data arrays. Therefore, when interpreting AFM images—especially without prior knowledge of the encoded message—users or administrators can focus on the most frequently occurring STV patterns to accurately read the intended information.

To ensure reliable data interpretation, certain criteria must be met during AFM analysis. First, interference from free STV molecules in solution must be excluded—only STV spots localized on the DDC structure are considered. Second, for each DDC structure, the total number of

STV spots is counted; pattern analysis is conducted only when the expected number of STVs is present. Finally, a pattern is classified as correct information only when the observed STV arrangement matches the expected data array.

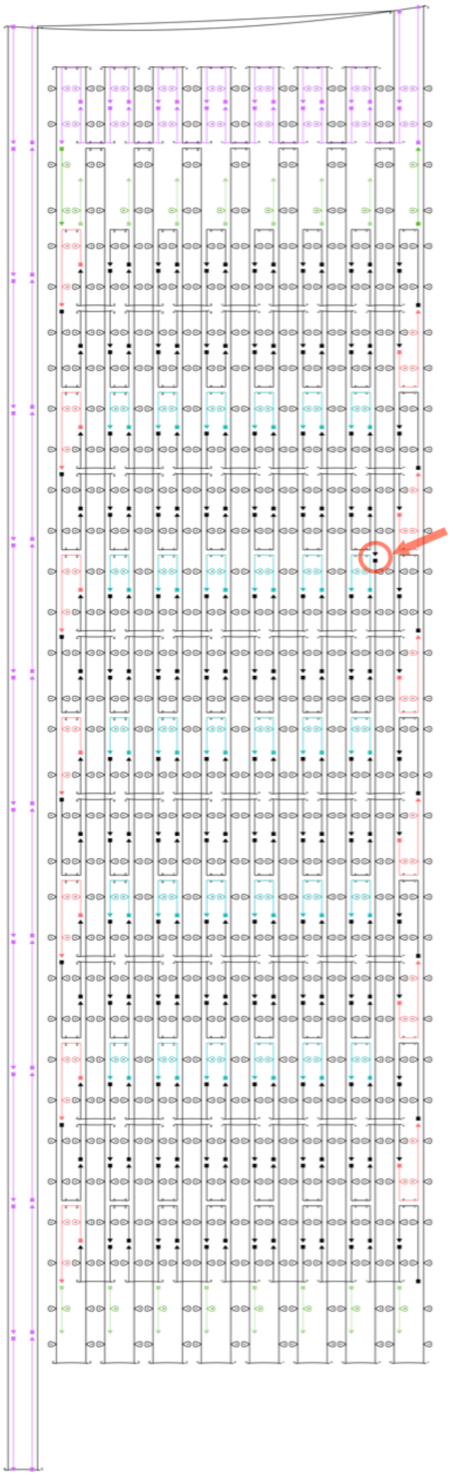

Figure S19: Layout of DDC-2 for user 2. Blue line: data array for user 2. The breakpoint of the scaffold strand (indicated by the red circle and arrow) differs from that in DDC-1. In caDNAno, scaffold sequences are generated starting from the breakpoint, and staple sequences are automatically assigned based on complementarity. Consequently, altering the position of the scaffold breakpoint results in a complete change in the staple sequences. This design ensures the orthogonality of the invading strands between DDC-1 and DDC-2.

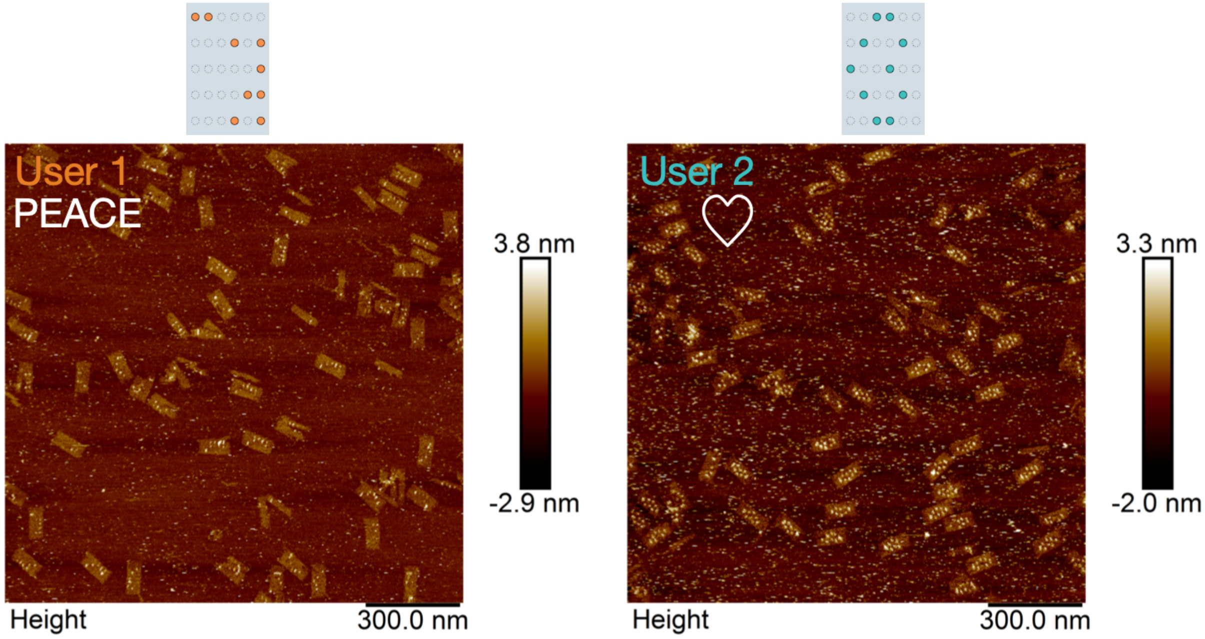

Figure S20: Representative AFM images of DDC structure with ciphertext in multi-user system.

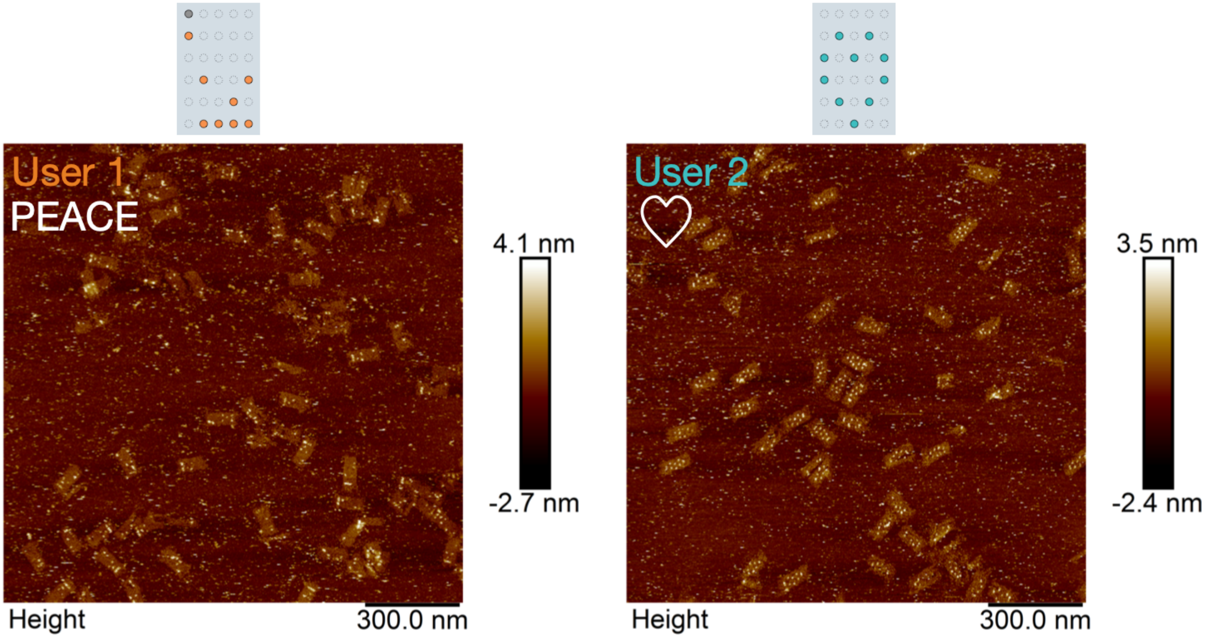

Figure S21: Representative AFM images of plaintext decoded by the admin key. User 1, "PEACE"; user 2, a heart-shaped icon.

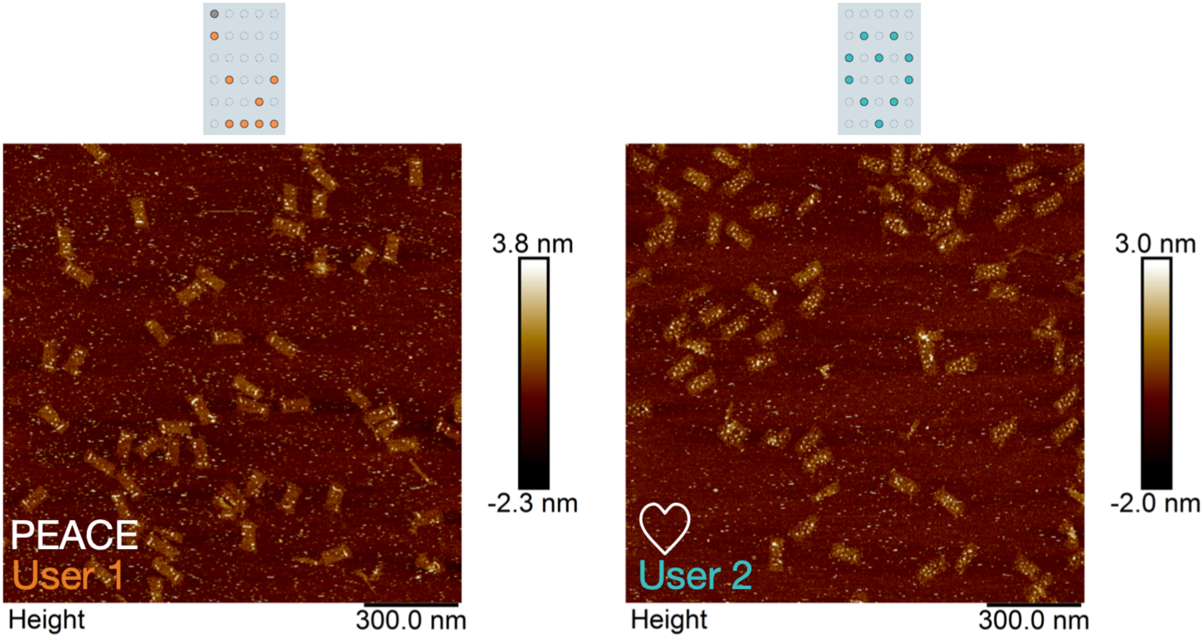

Figure S22: Representative AFM images of plaintext decoded by the user keys. User 1, "PEACE"; user 2, a heart-shaped icon.

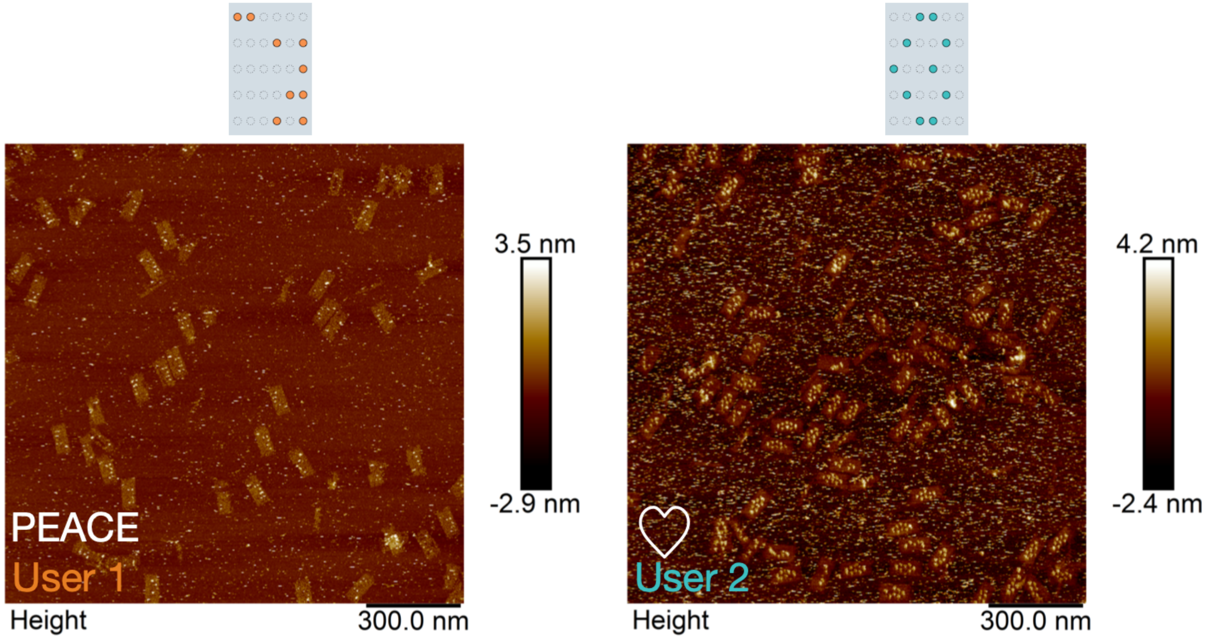

Figure S23: Representative AFM images of ciphertext that failed to decode using the mismatched user keys.

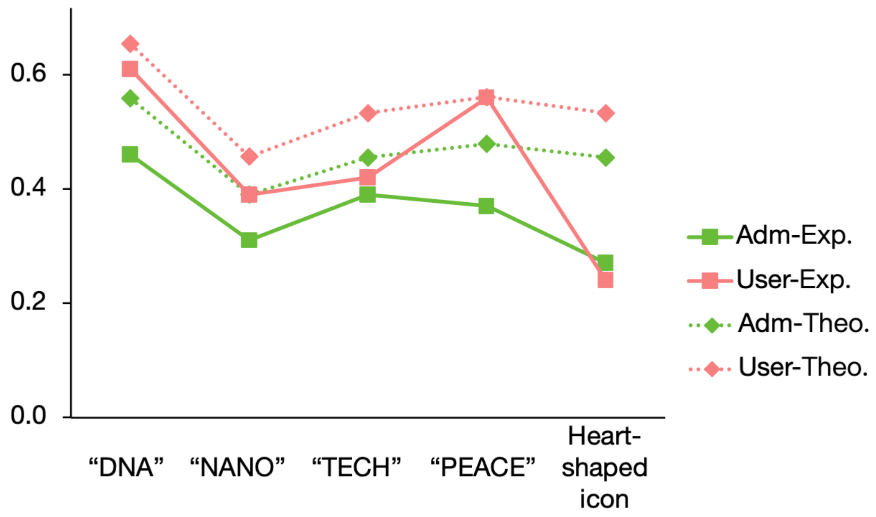

Figure S24: Line graph showing the yield of correct information retrieval across different samples. The green solid line represents the experimental yield of correct information retrieval using the administrator key, while the green dashed line indicates the corresponding theoretical value. The red solid line shows the experimental yield for samples accessed with the user key, and the red dashed line denotes the theoretical yield. In most cases, the experimental yields are slightly lower than the theoretical values, primarily due to characterization noise. Specifically, the AFM probe may take shadows near the STV points, leading to occasional misreading of the information. In the sample of heart-shaped icon, the deviation from theoretical values is more pronounced, attributed to missing address bits that render the reading direction indeterminate. Additionally, the sample exhibited a higher percentage of uncertain conformations compared to the others.

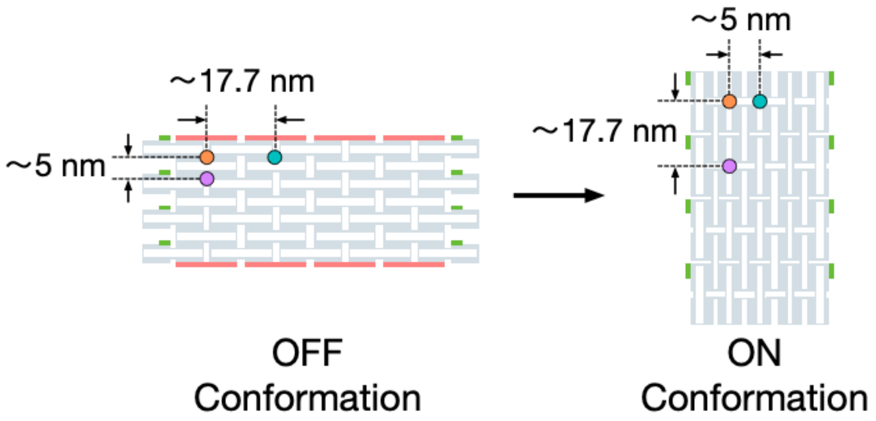

Figure S25: Diagram of the distances between adjacent STV sites in the data array before and after transformation. Note that the different colored dots in the diagram only represent the STVs in different positions.

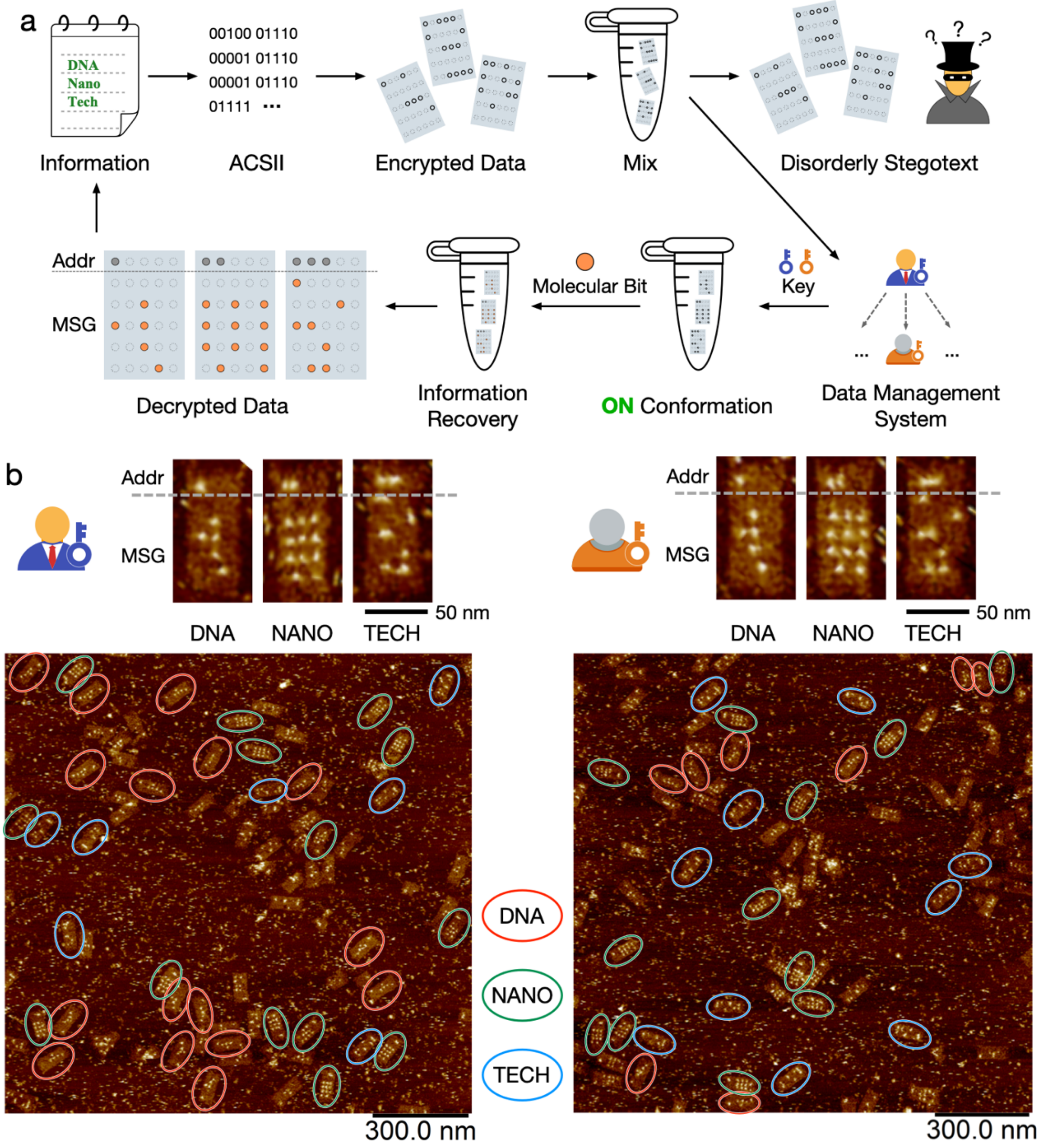

Figure S26: Simultaneous access to mixed three-word encoded messages. (a) Schematic flowchart of the three-word encoding and decoding process. Three distinct DDCs, each storing a different word, are mixed in a single tube. Before adding keys and the molecular bit, the data remains encrypted as stegotext on the DDCs. Upon the addition of admin or user keys, all three DDCs simultaneously switch to the ON conformation. Subsequent introduction of the molecular bit (STV) reveals the plaintext message on each DDC. (b) A complete three-word encrypted message is retrieved from an AFM image using either the admin or user key.

The three-word mixing experiment highlights improvements in both the information capacity and security of the message management system. By combining three DDCs in a single

sample, the information capacity per AFM characterization increases threefold. Moreover, because different words share the same DNA origami carrier structure, synthesized strands can be reused during encoding, enhancing the logical density of stored information.

The encryption strength against brute-force decryption via simple geometric transformations is also significantly improved. A two-dimensional rectangular DDC structure can yield four different data arrays through rotation. Flipping introduces two additional STV orientations, resulting in eight distinct configurations per DDC. When m distinct carriers are mixed in a tube, the total number of potential data arrays becomes 8^m. In this experiment, with $m = 3$, 512 possible arrays can be generated, rendering decryption by trial-and-error rotation or stretching infeasible. This scalability can be extended further. For instance, a 5-bit address space enables 32 unique address sequences; mixing 32 carriers would yield 8^{32} possible arrays—effectively unbreakable by simple transformation-based attacks. Only when the user or administrator introduces the appropriate key is the system brought into a readable state. At that point, the reader can confidently extract the correct information from the DDC structures exhibiting the highest proportion of the expected conformation. Without the key, an interceptor must rely on exhaustive trial-and-error to attempt decryption.

Two encryption layers enhance password security: a key layer and a steganographic layer. The user key consists of a specific set of DNA sequences, making it highly resistant to brute-force guessing, especially when the number and lengths of strands are unknown. The admin key derives its strength from precise regulation of polymerase reaction parameters. As shown in Figure S14, optimal polymerase concentration and duration must be finely tuned. Pre-agreed parameters according to DDC concentration between authorized users can prevent unauthorized access. Incorrect polymerase concentration or improper reaction timing risks disintegrating the information carrier, resulting in irreversible data loss.

The steganographic layer further contributes to security through its molecular conjugation mechanism. In this study, the STV–biotin binding pair was used to visualize the data array. However, users may adopt alternative molecular conjugations, such as sequence-programmable DNA dumbbell structures. The customizable molecular steganography adds another layer of protection, reinforcing the robustness of the overall encryption scheme.

2. Supporting Tables

Table S1. Conformation statistics of DDC before transformation (Figure 2c, left).

No.	OFF		ON		IC		Total
	Count	Percentage	Count	Percentage	Count	Percentage	
1	14	88%	1	6%	1	6%	16
2	30	88%	3	9%	1	3%	34
3	21	91%	1	4%	1	4%	23
4	8	67%	3	25%	1	8%	12
Sum	73	86%	8	9%	4	5%	85

Table S2. Conformation statistics of DDC after transformation using DNA strand set (Figure 2c, top right).

No.	OFF		ON		IC		Total
	Count	Percentage	Count	Percentage	Count	Percentage	
1	2	3%	74	95%	2	3%	78
2	2	14%	10	71%	2	14%	14
3	2	22%	6	67%	1	11%	9
Sum	6	6%	90	89%	5	5%	101

Table S3. Conformation statistics of DDC after transformation using polymerase (Figure 2c, bottom right).

No.	OFF		ON		IC		Total
	Count	Percentage	Count	Percentage	Count	Percentage	
1	1	9%	8	73%	2	18%	11
2	4	22%	13	72%	1	6%	18
3	3	16%	15	79%	1	5%	19
4	5	26%	14	74%	0	0%	19
5	3	17%	15	83%	0	0%	18
Sum	16	19%	65	76%	4	5%	85

Table S4. Yield statistics of the word “DNA”. (Figure 3b). EI, encoded information; DI_User, decoded information with user keys; DI_Adm, decoded information with admin key.

Sample		No.	OFF		ON				Uncertain		Total
					Transformed		CorrInfo				
			Ct.	Pct.	Ct.	Pct.	Ct.	Pct.	Ct.	Pct.	
DNA	EI	1	60	91%	2	3%	2	3%	4	6%	66
		2	44	83%	4	8%	3	6%	5	9%	53
		3	42	88%	3	6%	3	6%	3	6%	48
		4	33	92%	1	3%	1	3%	2	6%	36
		Sum	179	88%	10	5%	9	4%	14	7%	203
	DI_ User	1	1	13%	6	75%	5	63%	1	13%	8
		2	1	4%	20	83%	14	58%	3	13%	24
		3	5	20%	16	64%	14	56%	4	16%	25
		4	4	13%	25	83%	22	73%	1	3%	30
		5	4	14%	23	79%	16	55%	2	7%	29
		Sum	15	13%	90	78%	71	61%	11	9%	116
	DI _Adm	1	10	10%	77	80%	44	46%	9	9%	96
		2	6	9%	45	64%	31	44%	19	27%	70
		3	6	16%	26	68%	20	53%	6	16%	38
		4	9	15%	41	68%	26	43%	10	17%	60
		Sum	31	12%	189	72%	121	46%	44	17%	264

Table S5. Yield statistics of the word "NANO". (Figure 3b). EI, encoded information; DI_User, decoded information with user keys; DI_Adm, decoded information with admin key.

Sample		No.	OFF		ON				Uncertain		Total
					Transformed		CorrInfo				
			Ct.	Pct.	Ct.	Pct.	Ct.	Pct.	Ct.	Pct.	
NANO	EI	1	35	69%	5	10%	0	0%	11	22%	51
		2	21	51%	3	7%	0	0%	17	41%	41
		3	22	88%	2	8%	1	4%	1	4%	25
		4	7	78%	0	0%	0	0%	2	22%	9
		5	34	83%	4	10%	1	2%	3	7%	41
		Sum	119	71%	14	8%	2	1%	34	20%	167
	DI _User	1	8	10%	55	71%	25	32%	15	19%	78
		2	5	7%	65	89%	38	52%	3	4%	73
		3	7	9%	58	78%	22	30%	9	12%	74
		4	10	11%	78	83%	39	41%	6	6%	94
		Sum	30	9%	256	80%	124	39%	33	10%	319
	DI_ Adm	1	6	11%	38	69%	18	33%	11	20%	55
		2	8	10%	57	71%	22	28%	15	19%	80
		3	9	16%	28	48%	20	34%	21	36%	58
		Sum	23	12%	123	64%	60	31%	47	24%	193

Table S6. Yield statistics of the word "TECH". (Figure 3b). EI, encoded information; DI_User, decoded information with user keys; DI_Adm, decoded information with admin key.

Sample		No.	OFF		ON				Uncertain		Total
					Transformed		CorrInfo				
			Ct.	Pct.	Ct.	Pct.	Ct.	Pct.	Ct.	Pct.	
TECH	EI	1	47	76%	4	6%	4	6%	11	18%	62
		2	41	73%	2	4%	1	2%	13	23%	56
		3	45	79%	3	5%	2	4%	9	16%	57
		4	60	80%	4	5%	2	3%	11	15%	75
		5	48	80%	6	10%	4	7%	6	10%	60
		Sum	241	78%	19	6%	13	4%	50	16%	310
	DI_ User	1	5	12%	32	76%	19	45%	5	12%	42
		2	5	11%	38	81%	20	43%	4	9%	47
		3	5	10%	40	80%	16	32%	5	10%	50
		4	3	8%	30	77%	20	51%	6	15%	39
		Sum	18	10%	140	79%	75	42%	20	11%	178
	DI_ Adm	1	6	18%	26	76%	13	38%	2	6%	34
		2	7	14%	39	78%	21	42%	4	8%	50
		3	8	16%	35	69%	19	37%	8	16%	51
		4	10	19%	40	74%	20	37%	4	7%	54
		5	8	14%	42	75%	22	39%	6	11%	56
		6	8	17%	35	73%	20	42%	5	10%	48
		Sum	47	16%	217	74%	115	39%	29	10%	293

Table S7. Yield statistics of the word "PEACE". (Figure 4b & 4c). EI, encoded information; DI_User, decoded information with user keys; DI_Adm, decoded information with admin key.

Sample		No.	OFF		ON				Uncertain		Total
					Transformed		CorrInfo				
			Ct.	Pct.	Ct.	Pct.	Ct.	Pct.	Ct.	Pct.	
PEACE	EI	1	37	80%	5	11%	2	4%	4	9%	46
		2	35	83%	2	5%	1	2%	5	12%	42
		3	51	88%	2	3%	1	2%	5	9%	58
		4	42	86%	2	4%	1	2%	5	10%	49
		5	55	89%	3	5%	2	3%	4	6%	62
		Sum	220	86%	14	5%	7	3%	23	9%	257
	DI_ User	1	5	11%	37	84%	28	64%	2	5%	44
		2	5	9%	46	85%	30	56%	3	6%	54
		3	5	13%	32	82%	20	51%	2	5%	39
		4	6	14%	35	81%	22	51%	2	5%	43
		Sum	21	12%	150	83%	100	56%	9	5%	180
	DI_ Adm	1	8	24%	23	68%	11	32%	3	9%	34
		2	7	20%	22	63%	12	34%	6	17%	35
		3	4	11%	27	75%	15	42%	5	14%	36
		4	4	15%	18	67%	11	41%	5	19%	27
		Sum	23	17%	90	68%	49	37%	19	14%	132
	Mis-match	1	39	76%	3	6%	1	2%	9	18%	51
		2	48	86%	5	9%	2	4%	3	5%	56
		3	27	75%	4	11%	2	6%	5	14%	36
		4	35	73%	3	6%	2	4%	10	21%	48
		Sum	149	78%	15	8%	7	4%	27	14%	191

Table S8. Yield statistics of the heart-shaped icon. EI, encoded information; DI_User, decoded information with user keys; DI_Adm, decoded information with admin key.

Sample		No.	OFF		ON				Uncertain		Total
					Transformed		CorrInfo				
			Ct.	Pct.	Ct.	Pct.	Ct.	Pct.	Ct.	Pct.	
Heart	EI	1	49	84%	3	5%	3	5%	6	10%	58
		2	48	72%	2	3%	2	3%	17	25%	67
		3	37	65%	3	5%	2	4%	17	30%	57
		4	29	73%	2	5%	0	0%	9	23%	40
		Sum	163	73%	10	5%	7	3%	49	22%	222
	DI_ User	1	8	17%	31	66%	9	19%	8	17%	47
		2	8	13%	39	63%	10	16%	15	24%	62
		3	7	15%	30	65%	15	33%	9	20%	46
		4	9	14%	43	67%	18	28%	12	19%	64
		Sum	32	15%	143	65%	52	24%	44	20%	219
	DI_ Adm	1	8	26%	18	58%	10	32%	5	16%	31
		2	9	29%	18	58%	9	29%	4	13%	31
		3	9	20%	24	53%	13	29%	12	27%	45
		4	10	31%	16	50%	6	19%	6	19%	32
		Sum	36	26%	76	55%	38	27%	27	19%	139
	Mis- match	1	22	71%	0	0%	0	0%	9	29%	31
		2	45	80%	0	0%	0	0%	11	20%	56
		3	41	76%	3	6%	2	4%	10	19%	54
		4	51	80%	1	2%	0	0%	12	19%	64
		Sum	159	78%	4	2%	2	1%	42	20%	205

Table S9. Yield statistics of ON conformation in DDC structures with varied number of edge strands (Figure S4c).

Sample		Ct.	Pct.	Sum
VE-1	1	1	5%	21
	2	2	11%	18
	3	1	8%	12
	4	0	0%	15
	5	3	8%	36
	6	3	12%	25
	7	2	11%	19
	Sum	12	8%	146
VE-2	1	14	61%	23
	2	12	63%	19
	3	7	47%	15
	4	9	41%	22
	5	6	50%	12
	Sum	48	53%	91
VE-3	1	14	74%	19
	2	10	71%	14
	3	13	81%	16
	4	29	78%	37
	Sum	66	77%	86
VE-4	1	17	81%	21
	2	20	91%	22
	3	22	81%	27
	4	21	81%	26
	Sum	80	83%	96
VE-5	1	18	90%	20
	2	7	78%	9
	3	32	89%	36
	4	27	96%	28
	Sum	84	90%	93
VE-6	1	18	90%	20
	2	25	96%	26
	3	19	100%	19
	4	30	94%	32
	Sum	92	95%	97

REFERENCES

1. Fan, S., Wang, D., Cheng, J., Liu, Y., Luo, T., Cui, D., Ke, Y. and Song, J. (2020) Information coding in a reconfigurable DNA origami domino array. *Angew. Chem., Int. Ed.*, **59**, 12991-12997.
2. Song, J., Li, Z., Wang, P., Meyer, T., Mao, C. and Ke, Y. (2017) Reconfiguration of DNA molecular arrays driven by information relay. *Science*, **357**, eaan3377.
3. Chen, K., Xu, F., Hu, Y., Yan, H. and Pan, L. (2022) DNA kirigami driven by polymerase-triggered strand displacement. *Small*, **18**, 2201478.
4. Chen, K., Xie, C., Chen, Z., Wang, S., Hu, Y., Xu, F. and Pan, L. (2023) Presketched DNA origami canvas for polymerase-driven DNA kirigami. *ACS Nano*, **17**, 17265-17272.
